# Peritransplant glucocorticoids redistribute donor T cells to the bone marrow and prevent relapse after haploidentical SCT

**DOI:** 10.1172/jci.insight.153551

**Published:** 2021-11-22

**Authors:** Takayuki Inoue, Motoko Koyama, Katsuji Kaida, Kazuhiro Ikegame, Kathleen S. Ensbey, Luke Samson, Shuichiro Takahashi, Ping Zhang, Simone A. Minnie, Satoshi Maruyama, Shinichi Ishii, Takashi Daimon, Takahiro Fukuda, Hirohisa Nakamae, Takahide Ara, Yumiko Maruyama, Ken Ishiyama, Tatsuo Ichinohe, Yoshiko Atsuta, Bruce R. Blazar, Scott N. Furlan, Hiroyasu Ogawa, Geoffrey R. Hill

**Affiliations:** 1 Clinical Research Division, Fred Hutchinson Cancer Research Center, Seattle, Washington, USA.; 2 Division of Hematology, Department of Internal Medicine, Hyogo College of Medicine, Hyogo, Japan.; 3 Department of Hematology-Oncology, Chiba Cancer Center, Chiba, Japan.; 4 Division of Hematology, Kobe University Graduate School of Medicine, Hyogo, Japan.; 5 Department of Biostatistics, Hyogo College of Medicine, Hyogo, Japan.; 6 Department of Hematopoietic Stem Cell Transplantation, National Cancer Center Hospital, Tokyo, Japan.; 7 Department of Hematology, Osaka City University Hospital, Osaka, Japan.; 8 Department of Hematology, Hokkaido University Hospital, Hokkaido, Japan.; 9 Department of Hematology, University of Tsukuba Hospital, Ibaraki, Japan.; 10 Department of Hematology, Kanazawa University Hospital, Ishikawa, Japan.; 11 Department of Hematology and Oncology, Research Institute for Radiation Biology and Medicine, Hiroshima University, Hiroshima, Japan.; 12 Japanese Data Center for Hematopoietic Cell Transplantation, Tokyo, Japan.; 13 Department of Healthcare Administration, Nagoya University Graduate School of Medicine, Nagoya, Japan.; 14 Division of Blood and Marrow Transplantation, Department of Pediatrics, University of Minnesota, Minneapolis, Minnesota, USA.; 15 Department of Hematology, Osaka Gyoumeikan Hospital, Osaka, Japan.; 16 Division of Medical Oncology, University of Washington, Seattle, Washington, USA.

**Keywords:** Transplantation, Bone marrow transplantation, Cell migration/adhesion, Stem cell transplantation

## Abstract

Patients with acute leukemia who are unable to achieve complete remission prior to allogeneic hematopoietic stem cell transplantation (SCT) have dismal outcomes, with relapse rates well in excess of 60%. Haplo-identical SCT (haplo-SCT) may allow enhanced graft-versus-leukemia (GVL) effects by virtue of HLA class I/II donor-host disparities, but it typically requires intensive immunosuppression with posttransplant cyclophosphamide (PT-Cy) to prevent lethal graft-versus-host disease (GVHD). Here, we demonstrate in preclinical models that glucocorticoid administration from days –1 to +5 inhibits alloantigen presentation by professional recipient antigen presenting cells in the gastrointestinal tract and prevents donor T cell priming and subsequent expansion therein. In contrast, direct glucocorticoid signaling of donor T cells promotes chemokine and integrin signatures permissive of preferential circulation and migration into the BM, promoting donor T cell residency. This results in significant reductions in GVHD while promoting potent GVL effects; relapse in recipients receiving glucocorticoids, vehicle, or PT-Cy was 12%, 56%, and 100%, respectively. Intriguingly, patients with acute myeloid leukemia not in remission who received unmanipulated haplo-SCT and peritransplant glucocorticoids also had an unexpectedly low relapse rate at 1 year (32%; 95% CI, 18%–47%) with high overall survival at 3 years (58%; 95% CI, 38%–74%). These data highlight a potentially simple and effective approach to prevent relapse in patients with otherwise incurable leukemia that could be studied in prospective randomized trials.

## Graphical abstract

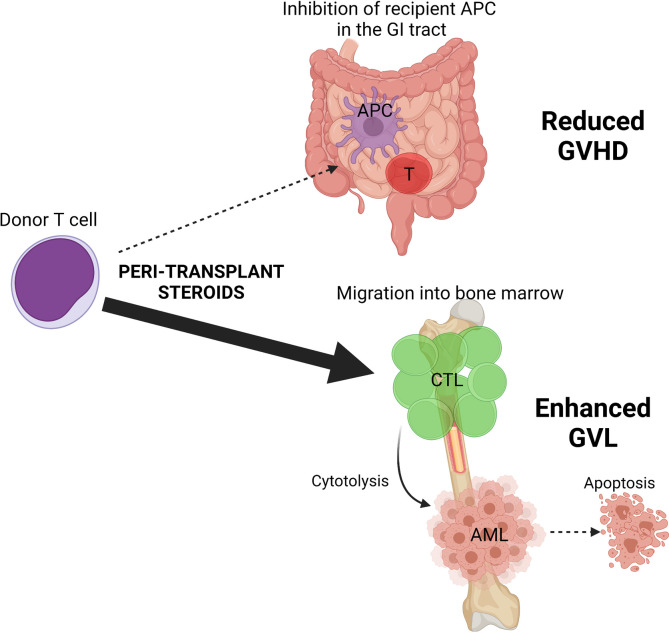

## Introduction

Allogeneic hematopoietic stem cell transplantation (allo-SCT) is a curative therapy for high-risk hematological malignancies, most commonly acute leukemia and myelodysplasia. The therapeutic efficacy lies in the immunological graft-versus-leukemia effects (GVL) in which donor T cells and NK cells play major roles in eradicating leukemic cells typically residing within the BM. The propensity for long-term disease-free survival is predicated on patients entering allo-SCT in complete remission (CR), including the absence of measurable residual disease by sensitive molecular (e.g., next-generation sequencing) and cellular (e.g., flow cytometry) techniques. Although conventional HLA-matched SCT leads to long-term survival beyond 55%–65% in patients with acute myeloid leukemia (AML) in CR (1, 2), patients who are not in morphological CR at the time of transplantation have a dismal outcome, with relapse in excess of 60% (3, 4) and survival at 3 years of less than 20% (5). These patients are, therefore, not usually viewed as transplant candidates.

The potency of the GVL effect is closely associated with graft-versus-host disease (GVHD), the major procedural limitation of allo-SCT, in which alloreactive donor T cells attack normal recipient tissues. To date, meaningful separation of GVHD and GVL has been met with limited success, and approaches to prevent GVHD have focused primarily on broad immune pharmacological suppression that concurrently inhibit GVL (6, 7). Recently, the ability to safely transplant across broad MHC barriers (e.g., across a haplotype in haploidentical SCT [haplo-SCT]) has been made possible by the use of posttransplant cyclophosphamide (PT-Cy), which can delete alloreactive T cells while sparing regulatory T cell responses (8). The use of HLA class I and II mismatched grafts in haplo-SCT provides a theoretical advantage in relation to the strength of the GVL response, since it greatly increases the spectrum of target alloantigens on residual leukemia. However, to date, the strength of GVL responses after haplo-SCT with PT-Cy–based immune suppression is debated and lacks definitive prospective controlled data relative to MHC-matched allo-SCT. Thus, as in matched unrelated donor transplantation, patients receiving haplo-SCT are generally restricted to those in CR. Interestingly, preclinical models of haplo-SCT using PT-Cy demonstrate a profound attenuation of GVL effect accompanied by rapid relapse (9), likely reflecting the aggressive nature and relatively high doses of the AML infused in these systems.

Clinically, GVHD within the gastrointestinal (GI) tract is the principal determinant of transplant-related mortality and is initiated by a network of alloantigen presentation by professional and nonprofessional antigen presenting cells (APC) that prime incoming donor T cells in the GI tract and related primary lymphoid structures (10, 11). The activated donor T cells become expanded in the lymphoid tissues and are thought to traffic to the target intestine through integrin/chemokine interactions (12, 13). In contrast, GVL is associated with donor T cell responses recognizing allogeneic, hematopoietic, and leukemia-specific antigens expressed on leukemia cells (14–16). Given that leukemia precursor cells reside in the BM, alloantigen-reactive donor T cells presumably mediate GVL and GVHD at spatially distinct sites: the BM and the GI tract, respectively.

We have previously shown that peritransplant glucocorticoid (GC) administration is permissive of nonmyeloablative T cell–replete haplo-SCT in high-risk patients not in CR that is associated with an unexpectedly low rate of relapse (17–19). In the present study, we modeled the effect of peritransplant glucocorticoids on GVHD and GVL in preclinical systems, focusing on T cells within the GI tract and BM. We confirmed clear reduction of donor T cell priming in the GI tract and GVHD following GC treatment, an effect mediated by recipient APC. In contrast, GC treatment enhanced GVL by promoting CD8^+^ T cell trafficking into the BM and long-term T cell residence. We also noted unexpectedly high survival as a result of reduced relapse in patients with AML who underwent haplo-SCT with GC-based GVHD prophylaxis relative to a similar group of patients receiving standard PT-Cy–based immune suppression. Thus, early steroid therapy appears to spatially modify donor T cell migration and expansion to inhibit GVHD in the GI tract while promoting potent GVL responses in the BM.

## Results

### GCs attenuate GVHD and permit effective GVL responses in preclinical haploidentical BMT models.

**Figure 1:**
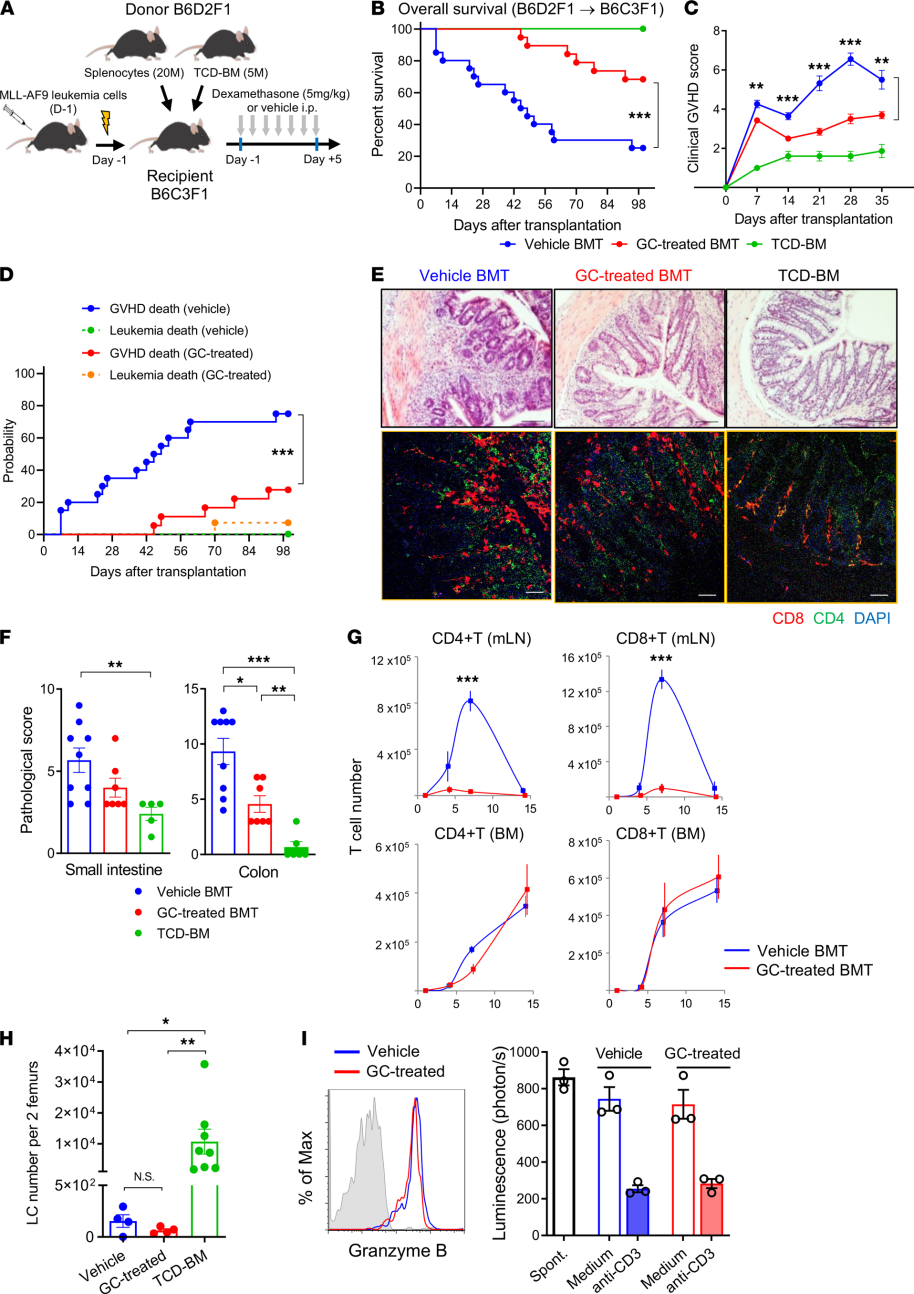
Glucocorticoids attenuate GVHD and permit effective GVL responses. B6C3F1 mice were transplanted with 5 × 10^6^ BM and 2 × 10^7^ splenocytes from B6D2F1 with or without glucocorticoid (GC) treatment. B6C3F1 background MLL/AF9-transduced leukemia cells were transplanted with T cell replete grafts (not TCD). (**A**) Haploidentical BMT model. (**B** and **C**) Overall survival by Kaplan-Meier analysis and clinical GVHD scores (compared by Student’s *t* test at the indicated time points) of the T cell replete groups. Results are combined from 2–3 experiments, 6–20 mice per group. (**D**) Competing risk analysis of GVHD versus leukemia death. (**E**) Representative H&E-stained sections (scale bars: 100 μm, upper panels) and immunofluorescent staining (scale bars: 50 μm, lower panels) of colon at day 14. (**F**) GVHD histopathology scores in the small intestine and colon. Results combined from 2 experiments (*n* = 5–9 per group). Multiple comparison by 1-way Welch ANOVA (colon) or Kruskal-Wallis test (small intestine). (**G**) Kinetics of donor CD4^+^ and CD8^+^ T cell numbers in the mLN (upper) and BM (lower panels) at day 4, 7 and 14 after allogeneic BMT (*n* = 6 per group from 3 experiments). Comparisons by Student’s *t* test with Welch’s modification. (**H**) Leukemia cell (LC) numbers per 2 recipient femurs at day 14 (*n* = 4–8 per group from 2 experiments). Multiple comparison by Kruskal-Wallis test. (**I**) Representative histogram of granzyme B expression (blue, vehicle; red, GC-treated) in donor CD44^+^CD8^+^ T cells at day 10 BM from vehicle- or GC-treated recipients (IgG isotype control in gray). Pooled donor-type H-2K^d+^CD44^+^CD8^+^ T cells were sorted by FACS at day 10 and cocultured with luciferase-expressing leukemic cells with or without soluble anti-CD3ε. Lytic activity as described in Methods. Data combined from 2 experiments. Data are presented as mean ± SEM. *P < 0.05, **P < 0.01; ***P < 0.001.

We sought to understand the immunological effects of peritransplant GC administration in robust preclinical models where transplant variables could be tightly controlled. We first utilized a haploidentical BM transplant (BMT) model (B6D2F1 [H-2^b/d^] B6C3F1 [H-2^b/k^]), in which lethally irradiated B6C3F1 mice were transplanted with splenocytes and T cell–depleted (TCD) BM cells from B6D2F1 mice and recipient-type (B6C3F1) MLL-AF9 AML. BMT recipients were then treated with daily dexamethasone (referred to as GC treated) or methyl–β-cyclodextrin (referred to as vehicle) from day –1 to +5 after transplantation (Figure 1A). Both groups achieved full donor chimerism within 14 days (data not shown). GC treatment improved transplant overall survival from 25% to 68% at day 100 (*P* = 0.0012, Figure 1B) with significant reductions in clinical GVHD scores (Figure 1C). We also determined the impact of GC on the GVL effect. The cumulative incidence analysis of GVHD death and leukemia death by competing risk analysis demonstrated that the majority of GC-treated recipients survived GVHD compared with control recipients (75% versus 26% at day 100, *P* = 0.0007; Figure 1D), while AML progression was equally suppressed in both groups (0% versus 5% at day 100, *P* = 0.3132; Figure 1D). Semiquantitative histopathological analysis demonstrated that GC-treated recipients had significantly reduced GVHD in the colon with reduced T cell infiltration compared with vehicle-treated recipients (Figure 1, E and F). We noted that donor CD4^+^ and CD8^+^ T cell infiltration was attenuated in the colon (Figure 1E, lower panels) in GC-treated recipients and was dramatically ablated in the gut-draining mesenteric lymph nodes (mLN) (Figure 1G, upper panels). In contrast, T cell expansion in the BM was comparable between the vehicle and GC-treated recipients (Figure 1G, lower panels). In conjunction with donor T cell expansion in the BM, leukemia cells in BM from GC-treated mice at day 14 were low and similar or lower in numbers to the vehicle control mice (Figure 1H). Finally, donor-type CD44^+^CD8^+^ effector T cells purified from the GC-treated BM cells expressed granzyme B and mediated equivalent killing on a per-cell basis to the vehicle-treated T cells in redirected cytolysis assays against leukemia cells (Figure 1I). These results demonstrate that GC treatment during the peritransplant period attenuates GVHD severity in the lower GI tract while preserving the GVL effect.

### GC treatment suppresses T cell activation and expansion in the mLN.

**Figure 2:**
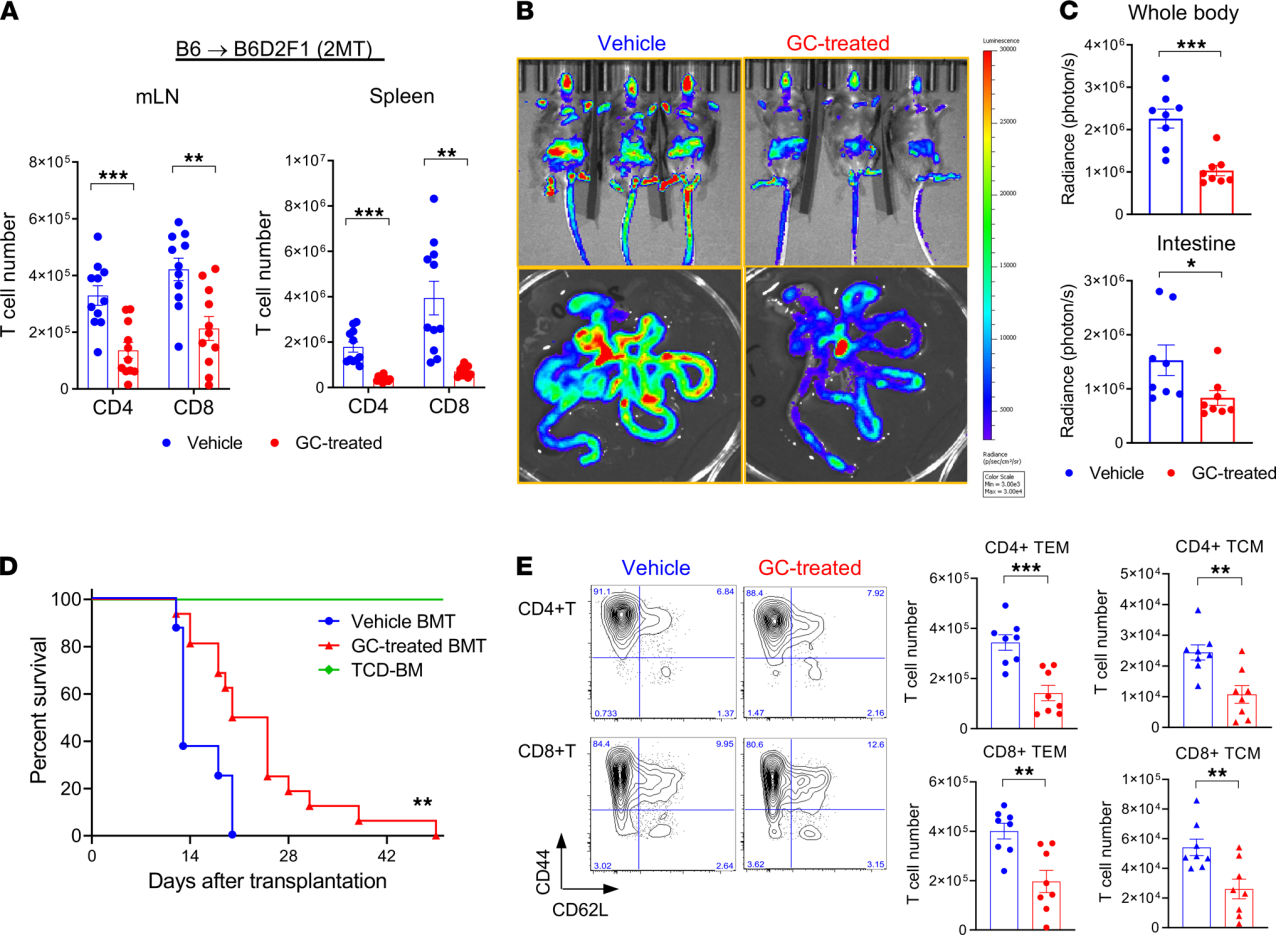
Glucocorticoid treatment suppresses T cell activation and expansion in the mLN. (**A**–**E**) B6D2F1 mice were transplanted with 5 × 10^6^ BM and 2 × 10^6^ (**A**–**C** and **E**) or 5 × 10^6^ (**D**) T cells from B6 mice with or without GC treatment (dexamethasone; 5 mg/kg/day i.p., days –1 to +5). (**A**) Donor-type CD4^+^ and CD8^+^ T cell numbers in the mLN (left) and spleen (right) at day 5 after BMT (combined from 2 experiments, *n* = 11 per group). (**B** and **C**) BLI of donor B6^luc+^ T cell expansion on day 7 after BMT. Representative BLI images (**B**) and BLI of whole body (top; ***P < 0.001) and intestine (bottom; *P < 0.05) (**C**). Results are combined from 2 experiments (*n* = 8 per group). (**D**) Survival by Kaplan-Meier analysis. Combined from 2 experiments with 6 (TCD) to 16 (T cell replete) mice per group. **P = 0.0047, vehicle- versus GC-treated BMT. (**E**) Representative flow cytometric plots of CD44 versus CD62L in donor CD4^+^ and CD8^+^ T cells in the mLN. CD44^+^CD62L^–^ (TEM) and CD44^+^CD62L^+^ (TCM) T cells in the mLN at day 5. Results are combined from 2 experiments with 8 mice per group. **P < 0.01; ***P < 0.001.

**Figure 3:**
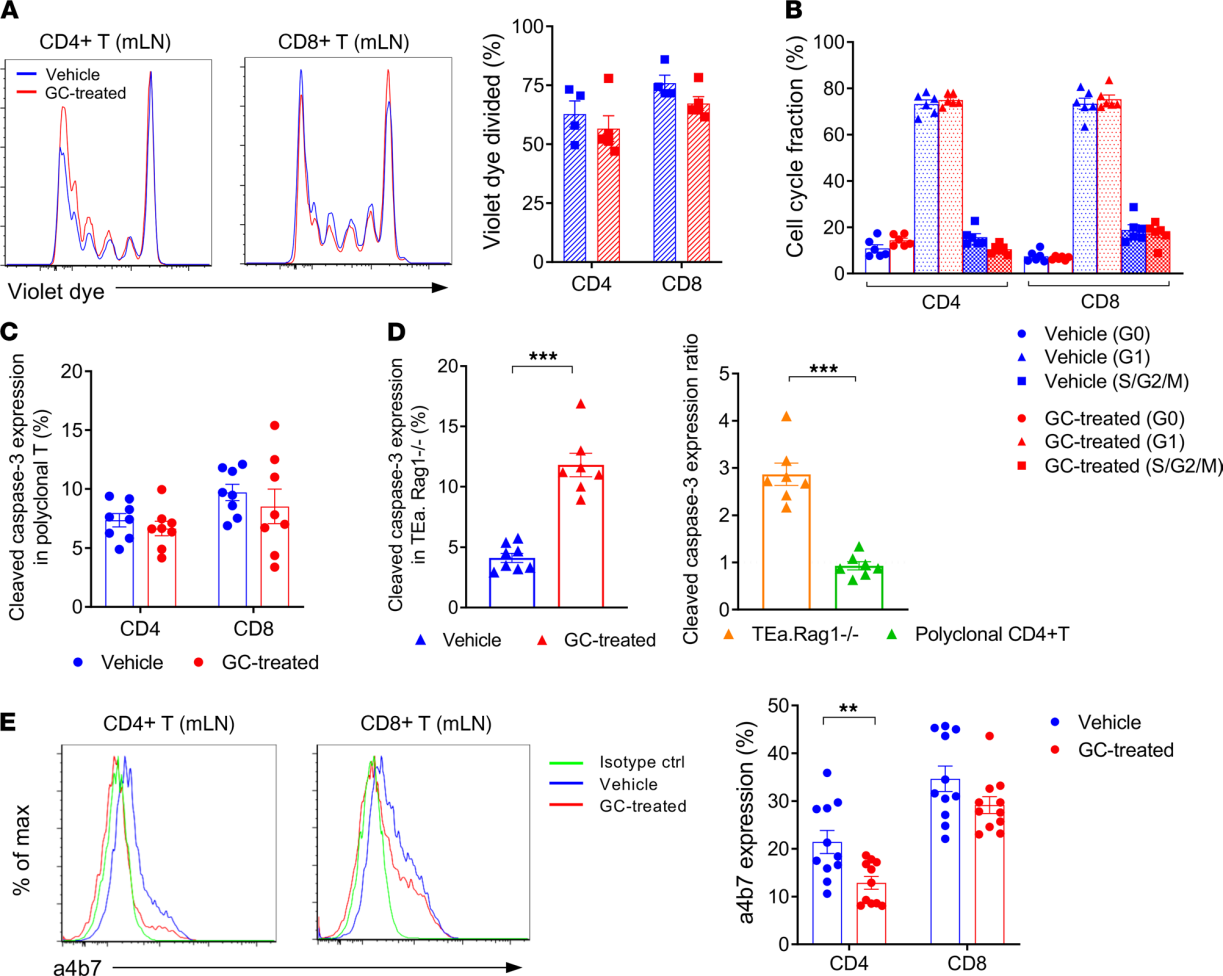
Glucocorticoid treatment has minimal effects on T cell proliferation of apoptosis in the mLN. (**A**, **B**, and **E**) B6D2F1 mice were transplanted with 5 × 10^6^ BM and 2 × 10^6^ from B6 mice with or without GC treatment. (**A**) Representative histograms and quantification of cell tracking violet dye–labeled CD4^+^ and CD8^+^ T cells in the mLN at day 3 (*n* = 4–5 per group from 2 experiments). (**B**) Cell cycle analysis of donor CD4^+^ and CD8^+^ T cells in the mLN are shown as evaluated by Ki-67 expression versus Hoechst dye staining at day 5 (*n* = 6 per group from 2 experiments). (**C** and **D**) A total of 4 × 10^3^ alloantigen-specific TEa.Rag1^–/–^ T cells (TEa) combined with congenic polyclonal 2 × 10^6^ WT B6 CD3^+^ T cells were transplanted with 5 × 10^6^ WT B6 BM into B6D2F1 mice. Cleaved caspase-3 expression of polyclonal donor CD4^+^ and CD8^+^ T cells (**C**) and TEa.Rag1^–/–^ cells (**D**, left) in the mLN at day 5. The ratios of caspase-3 expression in the TEa.Rag1^–/–^ and polyclonal CD4^+^ T cells from the GC-treated group (relative to vehicle-treated control) are shown (**D**, right). ***P < 0.001, *n* = 8 per group from 2 experiments. (**E**) Representative histogram plots (left) and quantified data (right) of integrin α4β7 expression on donor T cells in the vehicle- and GC-treated mLN at day 5. **P < 0.01, *n* = 11 per group from 3 experiments. Data are presented as mean ± SEM. Student’s *t* test with Welch’s modification.

We next undertook an alternative MHC-haploidentical BMT model (B6 [H-2^b^] B6D2F1 [H-2^b/d^]) in which recipients were transplanted with purified T cells and TCD BM cells to explore the mechanisms by which GC treatment modifies GVHD responses. GC treatment suppressed the expansion of both CD4^+^ and CD8^+^ T cells in the gut-draining mLN and secondary lymphoid organs at day 5 after BMT (Figure 2A). Bioluminescent imaging (BLI) demonstrated that GC treatment concurrently reduced the infiltration of luciferase-expressing B6 (B6^luc+^) T cells in the GI tract after BMT (Figure 2, B and C). Short-term GC treatment significantly attenuated GVHD mortality (Figure 2D). Analysis of mLN revealed that CD44^+^CD62L^–^ effector phenotype (effector memory T cells [TEM]) predominated among CD4^+^ and CD8^+^ T cells expanding at this site; however, GC treatment decreased the cell number of both CD44^+^CD62L^–^ TEM and CD44^+^CD62L^+^ central memory T cells (TCM) fractions (Figure 2E). No significant differences in the T cell proliferation were noted in the mLN to explain the reduction in numbers after GC treatment (Figure 3A). Cell-cycle analysis of donor T cells in the mLN further confirmed equivalent fractions within S/G2/M in GC and vehicle-treated controls (Figure 3B). We next explored the possibility that GC treatment was promoting apoptosis of alloreactive T cells. To compare alloantigen-specific T cells to polyclonal T cells, B6D2F1 mice were transplanted with BM and B6 WT (CD45.2^+^) and TCR transgenic T cells (TEa, CD45.1^+^CD4^+^) that react against recipient Eα-derived peptide presented in the MHC class II I-A^b^ molecule. Surprisingly, caspase-3 expression in both donor WT CD4^+^ and CD8^+^ T cells were comparable between the 2 groups (Figure 3C), and caspase 8 levels were also similar (Supplemental Figure 1; supplemental material available online with this article; https://doi.org/10.1172/jci.insight.153551DS1). However, GC treatment indeed promoted apoptosis in purely alloreactive TEa T cells (Figure 3D). Thus, while alloantigen-reactive T cells may be more susceptible to GC-induced apoptosis, neither T cell proliferation nor apoptosis could explain the discrepancy in the polyclonal T cell expansion between the 2 groups. In order to analyze whether GC treatment may alter T cell migration into the GI tract, we quantified the gut-homing integrin α4β7 expression and noted only subtle but significant reductions on CD4^+^ T cells (Figure 3E).

### GC effects on T cells in the GI tract are mediated indirectly via effects on alloantigen presentation.

**Figure 4:**
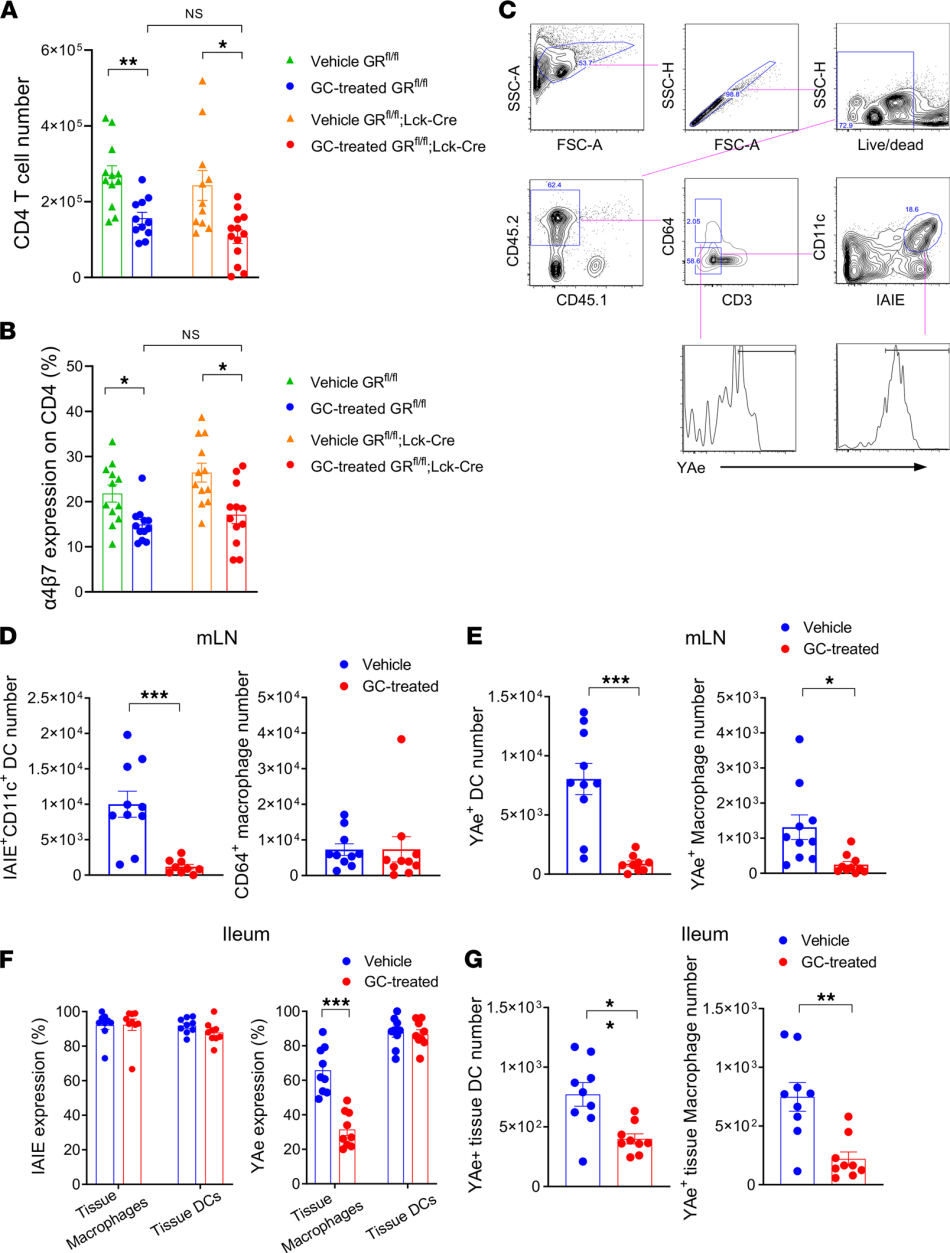
Glucocorticoid effects on T cells in the GI tract are mediated indirectly via effects on alloantigen presentation. (**A** and **B**) B6D2F1 mice received glucocorticoid receptor–deficient (GR-deficient; *lck^CRE^GR^fl/fl^* mice) or intact T cells (*GR^fl/fl^* littermates) with or without GC treatment. Donor CD4^+^ T cell numbers in the mLN and α4β7 expression on donor CD4^+^ T cells in the mLN at day 5. Results are combined from 3 experiments; *n* = 11–13 per group. Multiple comparison by 1-way Welch ANOVA test. (**C**–**G**) CD45.2^+^ B6D2F1 mice were transplanted with 5 × 10^6^ BM and 2 × 10^6^ T cells from CD45.1^+^ B6 mice with or without GC treatment. On day 1, mLN and ileum were analyzed. (**C**) Representative gating strategy is shown for the MHC II (IA/IE) and alloantigen (YAe) expression on recipient-type CD64^+^ macrophages and IA/IE^+^CD11c^+^ DCs in the mLN. (**D**) Absolute numbers of DCs (left) and macrophages (right) in the mLN. (**E**) The absolute numbers of alloantigen-presenting (YAe^+^) DCs (left) and macrophages (right) in the mLN. (**F**) Expression of IA/IE (left) and alloantigen presentation (YAe) (right) on recipient-type tissue-resident CD64^+^ macrophage and CD11c^+^ DCs in the terminal ileum. (**G**) The absolute numbers of YAe^+^ tissue macrophages (left) and DCs (right) in the terminal ileum. (**D**–**G**) Results are combined from 2–3 experiments. *n* = 10 per group. All quantified data are presented as mean ± SEM. Student’s *t* test with Welch’s modification. *P < 0.05; **P < 0.01; ***P < 0.001.

GCs have the capacity to affect a variety of immune cells, including those involved in innate immunity (20, 21). To determine whether GC administration exerted effects via direct or indirect signaling on the T cell, we transplanted GC receptor–deficient (GR-deficient) (*GR^fl/fl^*;*Lck-Cre* mice) or intact T cells (*GR^fl/fl^* littermates) with or without GC treatment. GC treatment significantly reduced donor CD4^+^ T cell expansion in the mLN, independently of their expression of the GR (Figure 4A), indicating that GC treatment modulates T cell expansion in the mLN via an intermediate cell. In conjunction with the reductions in T cell expansion, we noted that GC treatment also significantly downregulated the expression of gut-homing α4β7 integrin on CD4^+^ T cells in the mLN, again regardless of their GR expression (Figure 4B). We thereby hypothesized that GC treatment may modify the signals responsible for the initiation of alloreactive T cell expansion. We thus examined the alloantigen-presenting capacity of DCs and macrophages, which are involved in priming donor T cells in the GI tract (10, 11). We observed that GC treatment significantly reduced the number of recipient-type DCs in the mLN (Figure 4, C and D) and reduced the numbers of recipient DC and macrophages presenting alloantigen at this site (recipient-derived Eα peptide within MHC class II as confirmed by YAe antibody) (Figure 4E). While the proportions of macrophages and DCs expressing MHC class II in the ileum were not reduced by GC treatment (Figure 4F), the numbers of macrophages and DCs presenting alloantigen within the GI tract were also reduced by GC treatment (Figure 4G). Taken together, we demonstrate that peritransplant GC treatment modulated both the T cell expansion and α4β7 imprinting for donor CD4^+^ T cells mainly via indirect effects on recipient alloantigen presentation in the mLN and GI tract, which in turn correlated with the reduced infiltration of donor alloreactive T cells at this site.

### Peritransplant GC treatment enhances GVL.

**Figure 5:**
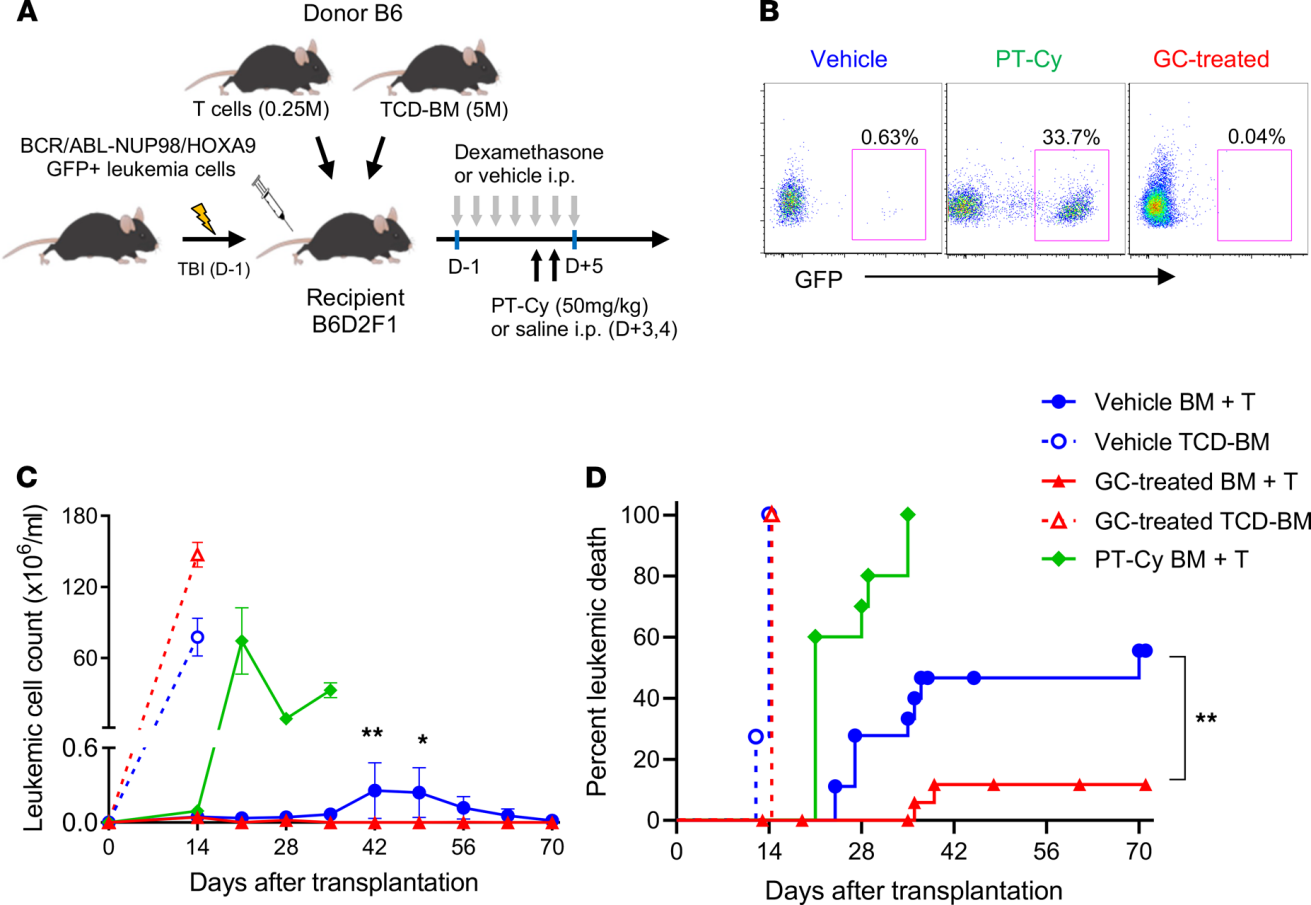
Peritransplant glucocorticoid treatment enhances GVL. (**A**) B6D2F1 mice were transplanted with 5 × 10^6^ BM, 0.25 × 10^6^ T cells, and 1 × 10^6^ BCR/ABL-NUP98/HOXA9 (GFP^+^) leukemia cells to compare GC treatment with PT-Cy conditioning (50 mg/kg, days +3 and +4). (**B**) Representative flow analysis of peripheral blood showed GFP^+^ leukemia cells at day 28 after transplant. (**C**) Leukemia cell numbers in the peripheral blood of each group compared by Student’s *t* test at the indicated time points. *P < 0.05; **P < 0.01. (**D**) Proportions of leukemia death. GC-treated versus vehicle-treated BMT recipients at day 70. **P = 0.0052 by log-rank analysis. Results are combined from 3 experiments with 10–20 mice per group.

We next utilized a second, aggressive primary AML (BCR/ABL-NUP98/HOXA9) system in order to further dissect the effects of GC treatment on GVL effects after BMT. In these experiments, we sought to compare GVL with or without GC treatment and PT-Cy that is routinely utilized clinically (Figure 5A). Recipient mice were transplanted with low T cell doses (0.25 × 10^6^) to allow quantification of the magnitude of GVL across groups. In these systems, PT-Cy profoundly attenuated GVL and was associated with rapid relapse in peripheral blood (Figure 5, B and C) and mortality, such that all animals died by day 40 (Figure 5D). In contrast, GC treatment resulted in low relapse rates and leukemia burdens in blood (Figure 5, B and C) with improved survival relative to vehicle-treated recipients (relapse rate: 12% versus 56% in GC versus vehicle-treated recipients at day 70, *P* = 0.0052; Figure 5D). When transplanted donor T cell doses were escalated (to 5 × 10^6^ per recipient) in the absence of leukemia, median survival in control versus GC-treated versus PT-Cy was 13 versus 22.5 versus > 42 days, respectively. Thus, PT-Cy provided superior protection from GVHD relative to GCs (*P* < 0.0001) but at the expense of profoundly impaired GVL.

### GC treatment promotes donor CD8^+^ T cell accumulation in the BM.

**Figure 6:**
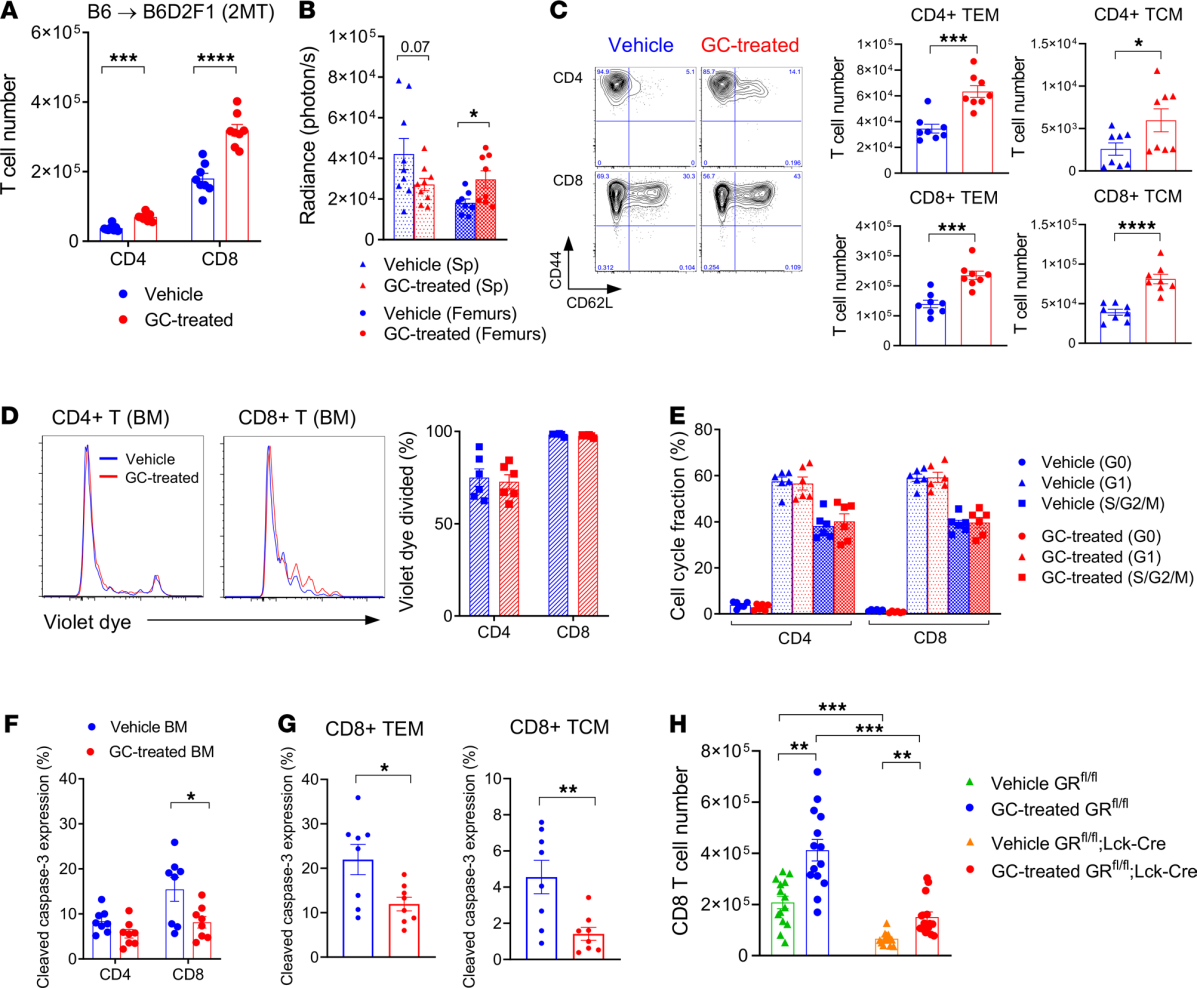
Glucocorticoid treatment promotes donor CD8^+^ T cell accumulation in the BM. B6D2F1 mice were transplanted with 5 × 10^6^ BM and 2 × 10^6^ T cells from B6 mice with or without GC treatment. (**A**) Donor CD4^+^ and CD8^+^ T cell numbers in the BM at day 5 after BMT. Results combined from 2 experiments, 8 mice per group. (**B**) BLI of donor B6^luc+^ T cells in the spleen and femurs at day 5 (*n* = 9 per group from 2 experiments). (**C**) Representative flow cytometric plots of CD44 versus CD62L in donor CD4^+^ and CD8^+^ T cells, and quantified numbers of CD44^+^CD62L^–^ (TEM) and CD44^+^CD62L^+^ (TCM) cells in the BM at day 5 (*n* = 8 per group from 2 experiments). (**D**) Representative histogram plots and quantification for violet dye dilution of CD4^+^ and CD8^+^ T cells in the BM at day 3 (*n* = 6 per group from 2 experiments). (**E**) Cell cycle fractions of donor T cells in the BM as evaluated by Ki-67 expression and Hoechst dye staining at day 5 (*n* = 6 per group from 2 experiments). (**F**) Cleaved caspase-3 expression in donor CD4^+^ and CD8^+^ T cells in the BM at day 5 (*n* = 8 per group from 2 experiments). (**G**) Cleaved caspase-3 expression in donor CD8^+^ T cells within TEM CD8 (left) and TCM CD8 (right) in the BM (*n* = 8 per group from 2 experiments). (**H**) B6D2F1 mice received glucocorticoid receptor–deficient (GR-deficient) (*lck^CRE^GR^fl/fl^* mice) or intact T cells (*GR^fl/fl^* littermates) with or without GC treatment. Donor CD8^+^ T cell numbers in the BM at day 5 (*n* = 13–14 per group). Results are combined from 3 experiments. Multiple comparison by 1-way Welch ANOVA test. *P < 0.05; **P < 0.01; ***P < 0.001; ****P < 0.0001. (**A**–**C**, **F**, and **G**) Student’s *t* test with Welch’s modification.

Given the enhanced GVL effect after GC treatment, we sought to understand the immunological consequences of this therapy within the recipient BM. In stark contrast to the effects seen in lymphoid organs and the GI tract, we noted that peritransplant GC treatment increased donor CD8^+^ T cell infiltration and, to a lesser extent, CD4^+^ T cell infiltration within the BM (Figure 6, A and B). Complete donor chimerism was achieved in the BM by day 14, regardless of GC exposure (data not shown). The absolute cell number of both effector (CD44^+^CD62L^–^) and central memory (CD44^+^CD62L^+^) fractions were significantly increased by GC treatment, although the former was numerically up to 10-fold higher (Figure 6C). In spite of this increase in T cell infiltration in the BM, there was no difference in proliferation of donor T cells at this site relative to control-treated recipients (Figure 6D). Consistent with this, cell cycle analysis indicated that the S/G2/M phase of donor T cells in the BM was similar in the 2 groups (Figure 6E). Interestingly, the proportions of donor apoptotic CD8^+^ T cells were decreased in the GC-treated recipients (Figure 6F), an effect seen in both effector and central memory (CM) donor CD8^+^ T cells (Figure 6G). We noted that the CM population had much lower proportions of apoptosis than the effector memory population (Figure 6G). We next transplanted GR-deficient T cells to examine the mechanisms of this GC effect in the BM and noted that GC treatment had both direct and indirect effects on donor T cell accumulation within the BM, but the direct effects on donor CD8^+^ T cells appeared to dominate (Figure 6H).

**Figure 7:**
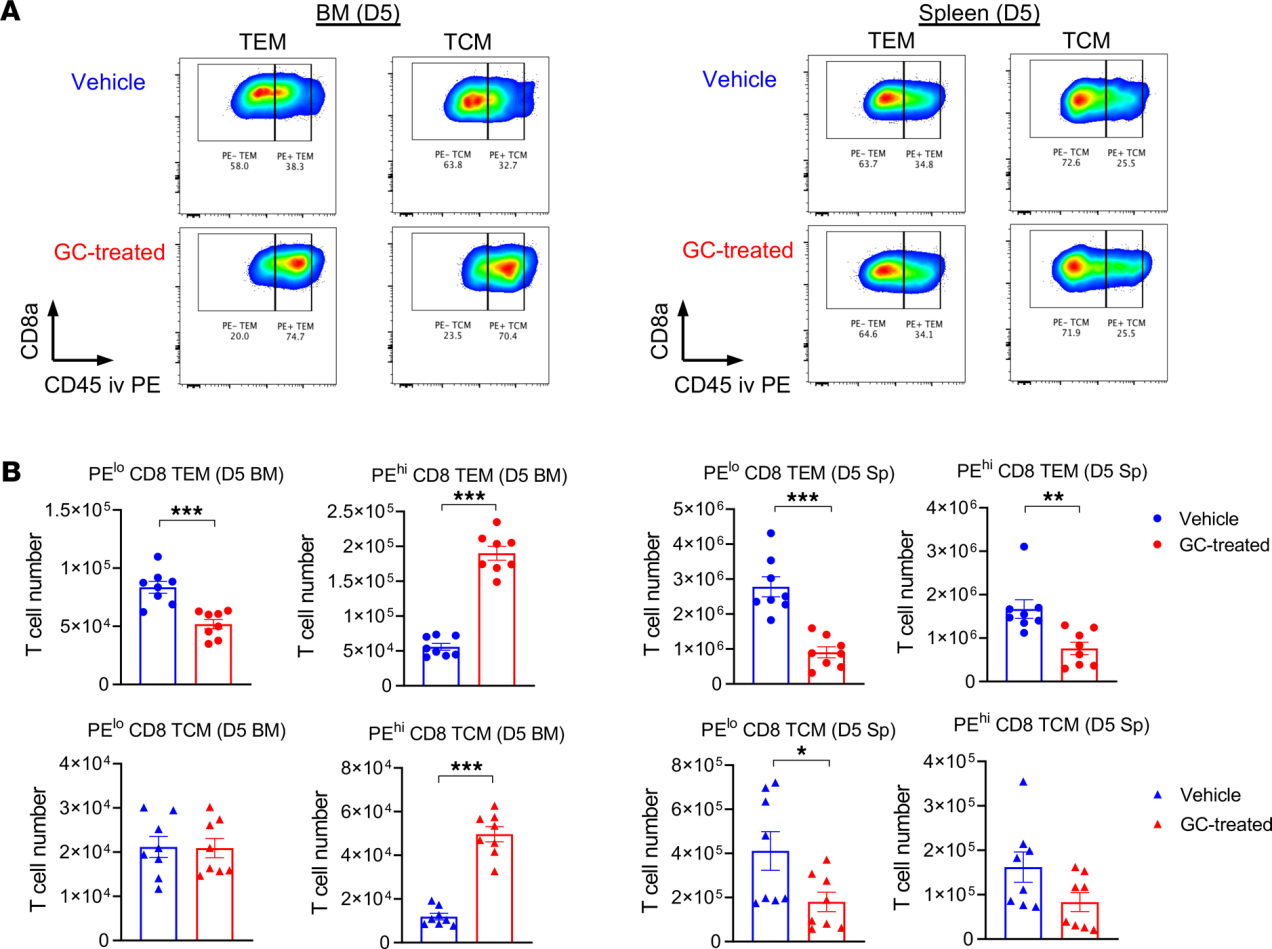
Glucocorticoid treatment promotes donor CD8^+^ T cell migration to the BM. (**A** and **B**) B6D2F1 mice were transplanted with 2 × 10^6^ purified B6.CD45.1^+^CD3^+^ T cells with or without GC treatment. On day 5 after transplantation, recipient mice were injected with a fluorescent-labeled anti-CD45 PE antibody by i.v. injection 5 minutes before euthanasia. (**A**) Representative FACS plots of CD45 expression on the gated donor CD8^+^ T cells in the BM (left) and spleen (right) from the vehicle- and GC-treated recipients at day 5 after transplantation. (**B**) Absolute numbers of CD45.1^+^ resident (PE low) and circulatory (PE high) CD8^+^ T cells with TEM and TCM phenotype in the BM and spleen (*n* = 8 per group, combined from 2 experiments). The fraction of peripheral blood–associated T cells was excluded. *P < 0.05; **P < 0.01; ***P < 0.001. All quantified data are presented as mean ± SEM. Student’s *t* test with Welch’s modification.

Finally, to understand whether GC treatment promoted donor T cell trafficking to the BM, we labeled intravascular T cells in vivo to distinguish between circulatory T cells and resident T cells in tissue (Figure 7A). These studies confirmed that GC treatment significantly increased the fraction and absolute numbers of donor CD8^+^ T cells circulating through the BM 5 days after BMT (Figure 7B). In contrast, the number of circulating T cells in the spleen, a secondary lymphoid organ, were reduced. These effects of steroids were not T cell restricted, since similar increases were seen in monocytes and neutrophils circulating in the BM (Supplemental Figure 2, A–D). While the numbers of resident donor CD8^+^ T cells in the BM were low early after transplant during GC treatment (Figure 7B), we noted increased numbers of donor T cells resident in the BM of the GC-treated recipients by day 27 after BMT (Supplemental Figure 2, E and F). Thus, GC treatment appeared to enhance GVL by directly increasing the circulation of donor T cells within the BM sinusoids at early time points after BMT, followed by accumulation within the BM parenchyma, where T cells putatively interact with leukemia that resides therein and mediate cytolysis.

**Figure 8:**
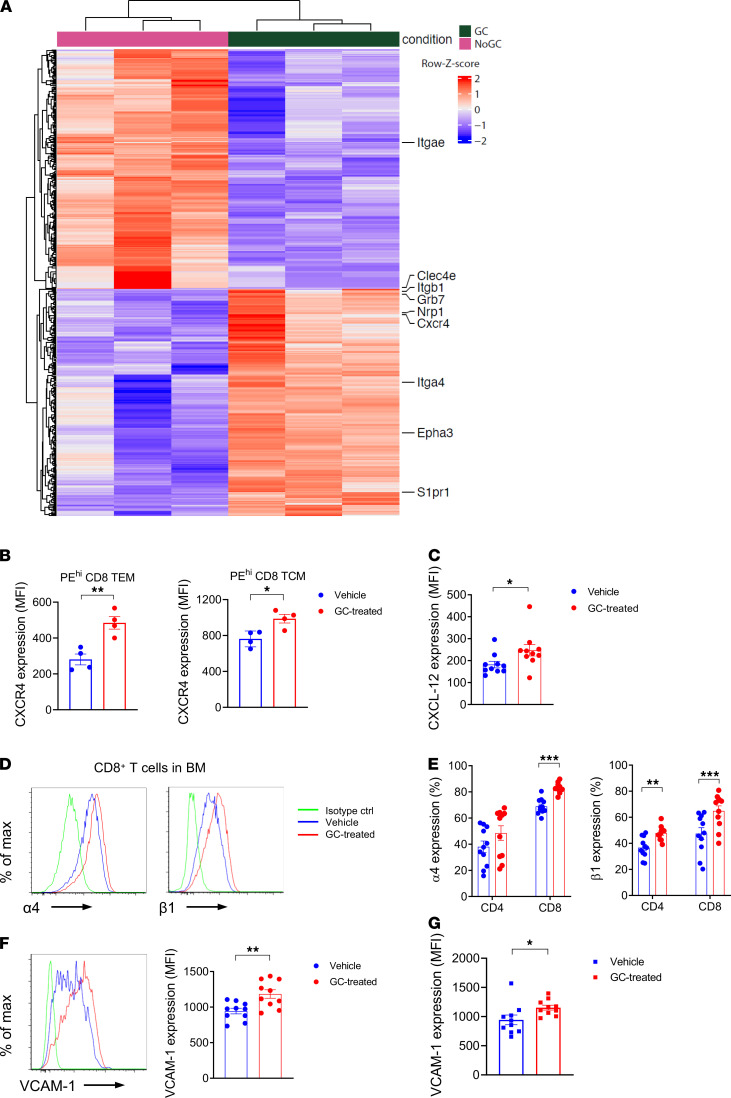
Glucocorticoids induce changes in migration and adhesion molecules to promote migration into the BM. (**A**) Bulk RNA-Seq of (in vivo CD45 labeled) circulating CD8^+^ TEM T cells in the BM at day 5 after BMT. Top differentially expressed genes (rows) in glucocorticoid-treated versus control animals (columns). Shown are the row-scaled data, which were subject to variance stabilized transformation. (**B**) CXCR4 expression on circulating TEM and TCM in the BM (*n* = 4 per group). (**C**) CXCL12 expression on BM CD45^–^CD31^–^TER119^–^Sca-1^+^ mesenchymal stromal cells (MSCs) at day 5 after BMT (*n* = 10–15 per group from 2 experiments). (**D**) Representative histogram plots of integrin α4 and β1 expression on donor CD8^+^ T cells in the BM at day 5. (**E**) Quantification of **D**. Results are combined from 3 experiments (*n* = 11 per group). (**F** and **G**) Representative histogram plots of VCAM-1 expression on MSCs (**F**) and CD45neg CD31+ TER119neg BM endothelial cells at day 5 after BMT and quantification (**G**) (*n* = 10 per group from 2 experiments). *P < 0.05; **P < 0.01; ***P < 0.001. Results are combined from 2–3 experiments. All quantified data are presented as mean ± SEM. Student’s *t* test with Welch’s modification.

In order to understand the direct effects of GCs on donor T cells responsible for this effect, we undertook bulk RNA-Seq on circulating CD8^+^ T cells after i.v. labeling with CD45 in the BM. This demonstrated the differential expression of a large number of molecules involved in cell homing and migration (Figure 8A). In particular, GC treatment was associated with reductions in molecules involved in homing to epithelial tissue such as CD103 (Itgae) and responses to damage-associated molecular pattern/pathogen-associated molecular pattern (DAMP/PAMP) signals (Clec4e). Conversely, molecules known to be involved in T cell homing to the BM such as CXCR4, S1P receptor 1 (S1pr1), and α4 integrin were increased in response to GC treatment. We confirmed that GC treatment enhanced the protein expression of CXCR4 by BM donor T cells (Figure 8B), together with its cognate ligand CXCL12 in BM stroma (Supplemental Figure 3 and Figure 8C). We also confirmed that GCs increased α4 and β1 integrins (Figure 8, D and E) that are known to preferentially promote T cell migration to the BM. In conjunction, we also noted increased expression of VCAM-1, the cognate ligand for α4β1, by BM mesenchymal cells (Supplemental Figure 3 and Figure 8F) and endothelial cells (Figure 8G). Together, these data highlight the capacity of peritransplant GCs as a simple and effective means to separate pathogenic GVHD reactions in the GI tract, while promoting beneficial GVL effects in the BM.

### Outcomes of haplo-SCT after GC-based immune suppression.

**Figure 9:**
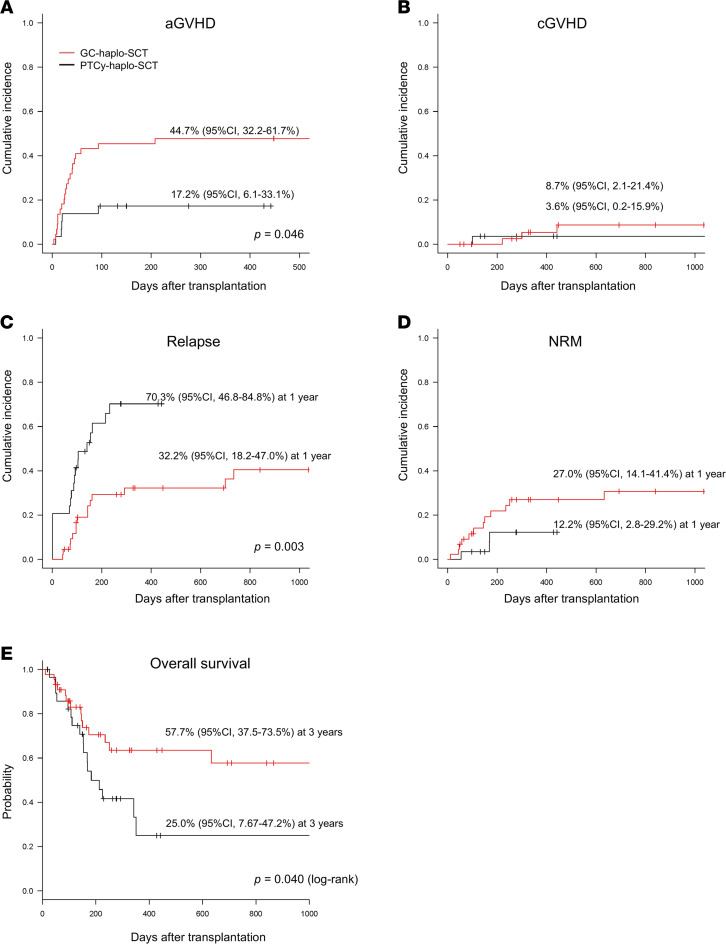
Outcomes of haploidentical SCT after glucocorticoid-based versus posttransplant cyclophosphamide–based immune suppression. (**A**) Cumulative incidence of grade II–IV GVHD. (**B**) Cumulative incidence of extensive chronic GVHD. (**C**) Cumulative incidence of relapse. (**D**) Cumulative incidence of NRM. (**E**) Overall survival by Kaplan-Meier estimates. Red and black lines indicate GC haplo-SCT and PT-Cy haplo-SCT, respectively.

We next studied outcomes in patients with AML not in morphological remission who received haplo-SCT. Forty-four patients underwent GC-based GVHD prophylaxis (methylprednisolone; 40 mg/day, starting from day –9) and 29 patients underwent a PT-Cy–based regimen and were extracted from the Japanese transplant registry (19, 20). Leukemia burdens were stratified into 3 groups by the proportions of blasts in the BM and PB before transplantation (Supplemental Table 1). Haplo-SCT recipients receiving GC-based immune suppression received significantly more CD34 cells and had improved rates of engraftment (Supplemental Figure 4, A and B, and Supplemental Table 2). The cumulative incidence of grade II–IV acute GVHD was 45% (95% CI, 32%–62%) for GC–haplo-SCT and 17% (95% CI. 6%–33%) for PT-Cy–haplo-SCT (Figure 9A). In multivariable analysis, a significantly higher incidence of acute GVHD was observed in the GC versus PT-Cy haplo-SCT (*P* = 0.046, Supplemental Table 2). The cumulative incidence of extensive chronic GVHD was similar: 8.7% (95% CI, 2%–21%) for GC haplo-SCT and 3.6% (95% CI, 0%–16%) for PT-Cy haplo-SCT (Figure 9B).

In multivariable Fine and Gray proportional hazards analysis, patients undergoing the GC haplo-SCT had a lower relapse rate than those undergoing PT-Cy haplo-SCT (*P* = 0.003, Supplemental Table 2); the cumulative incidence of relapse at 1 year was 32% (95% CI, 18%–47%) for GC haplo-SCT and 70% (95% CI, 47%–85%) for PT-Cy haplo-SCT (Figure 9C). The cumulative incidence of NRM at 1 year was 27% (95% CI, 14%–41%) for GC haplo-SCT and 12% (95% CI, 3%–30%) for PT-Cy haplo-SCT (Figure 9D). Consequently, recipients of GC haplo-SCT had a significantly higher overall survival (OS) at 3 years (58%; 95% CI, 38%–74%) compared with the PT-Cy recipients (25%; 95% CI, 8%–47%) (Figure 9E). In multivariate analysis, the risk of mortality was significantly lower in the recipients of GC haplo-SCT but was not associated with any other donor or recipient variables (hazard ratio [HR], 0.376, *P* = 0.041, Supplemental Table 3).

## Discussion

The separation of GVHD and GVL remains an overarching aspiration of allo-SCT but has been difficult to achieve in clinical practice. Current approaches to minimize relapse include the use of myeloablative conditioning, the use of peripheral blood stem cell (PBSC) and HLA-mismatched grafts, and the administration of posttransplant leukemia targeted therapy (e.g., kinase inhibitors, hypomethylating agents, or gene modified T cells such as chimeric antigen receptor [CAR] and TCR transgenic T cells) (22). Recently, the presence of measurable residual disease before or in the early period after transplant has been shown to be highly predictive of relapse (23). Indeed, the presence of morphological acute leukemia prior to transplant, particularly following relapse, has long been known to represent a largely incurable state, even after myeloablative PBSC transplantation (3). Overall survival in patients not in remission receiving haplo-SCT are also reported to be less than 25% at 2 years (24). Here, we demonstrate the ability of peritransplant GC administration to promote donor T cell migration and expansion, specifically in the BM with consequent augmented GVL — a scenario that also appears to be associated with high survival and low relapse rates in patients with active disease pretransplant.

GCs are known to act on T cells to dampen TCR signaling and cytokine expression. Some of the potent antiinflammatory actions of GCs occur by inhibiting the production of proinflammatory cytokines, integrin/chemokines, and other soluble mediators (20, 25). Utilizing T cell–specific GR depletion, we demonstrated that donor T cell suppression by GCs in the GI tract results from indirect signals rather than direct signaling within T cells. GC treatment, thus, decreased donor CD4^+^ T cell expansion and α4β7 integrin expression, independently of GR expression by the T cell. The profound reduction in recipient macrophage and DCs within the GI tract following GC administration is consistent with the fact that integrin α4β7 expression by donor T cells is driven by cognate antigen recognition (11). Here, we demonstrate that the indirect effect of GCs on T cells appears to be mediated by both quantitative and qualitative impacts on recipient APC in the GI tract, and this dominates any direct effect of GC on T cells, a result that was unexpected. We acknowledge that the effects of steroids on APC may also be direct or indirect and that the T cell repertoire may be altered in GR deficient T cells (26), an experimental variable that cannot be controlled for at this point in time. This GC effect putatively reflects enhanced APC apoptosis and reductions in PAMP-induced alloantigen presentation, consistent with the critical role of gut APCs in the initiation of lethal GVHD (11, 27, 28). The consequent rate of significant (grade II–IV) clinical GVHD in the GC-treated patients was acceptable in this high-risk patient group, though it was higher than seen after PT-Cy, where alloreactive T cell deletion in the context of retained Treg responses provide potent control of GVHD (8, 9). Importantly, however, the PT-Cy effect on GVHD occurred in the context of reduced leukemia-specific immunity and subsequent lower overall survival in these very high–risk patients.

In contrast, the GC effect in T cells within the BM are dependent on both direct and indirect signaling. Endogenous GC production at steady state is well established to modulate immunity via effects on leukocyte distribution and retention (29). Moreover, exogenous GC administration promotes the BM tropism of leukocytes, including T cells (30). Physiological GC functions to induce the redistribution of CD4^+^ T cells between peripheral blood and lymphoid organs through the upregulation of IL-7R/CXCR4 signaling in a diurnal fashion (31, 32). In these studies, GR signaling induced CXCR4 expression on T cells and expression of the cognate ligand CXCL12 in BM stroma that controlled T cell redistribution to the BM. CXCL12 signaling of CXCR4 on neutrophils augment VLA-4 (α4β1) adhesion to VCAM-1 in vitro, and this CXCL12/CXCR4 pathway may augment VLA-4 expression since blockade of both CXCR4 and α4 in vivo causes release of BM neutrophils in a synergistic fashion (33). Together, the α4β1/VCAM-1 adhesion pathway is critical in the retention and maturation-controlled release of leukocytes from the BM, while providing an important link between the CXCR4/CXCL12 signaling axis and the adhesion events that govern this process.

The Belkaid group recently reported that dietary restriction enhances GC levels to reduce memory T cell populations within secondary lymphoid organs and blood while enhancing their accumulation in BM. This effect was reproduced by the exogenous administration of GCs. This study demonstrated that this response was associated with profound remodeling of the BM compartment, with increases in adipocytes and T cell trophic factors and in erythropoiesis, as well as augmented CXCR4-CXCL12 and S1P-S1P1R interactions (34). These findings are consistent with our data after transplantation, where we demonstrated GR signals directly promote T cell circulation into the BM early after BMT, in association with upregulation of CXCR4 and α4β1 expression on CD8^+^ T cells together with the promotion of VCAM-1/CXCL12 expression on BM stroma indirectly. The data suggest also that the effects of GCs are highly dependent on the tissue in which the cells are residing, and GCs may, thus, not impact GVL at extramedullary sites. Given the differing cognate signals that a T cell sees within a tissue differ dramatically (27), this is perhaps not surprising and is an interesting avenue to explore in future studies. Together, these effects promoted potent GVL effects in preclinical systems on the basis of increased migration and subsequent accumulation in the BM parenchyma rather than apparent increases in cytolytic function on a per-cell basis.

The early administration of GCs was also associated with unexpectedly good outcomes in patients with AML receiving haplo-SCT while not in remission within Japan, but it must be noted that these data have significant limitations. Sample sizes are small and heterogenous, particularly in relation to conditioning regimens and the use of ATG in the GC group, albeit at the lowest dose permissive of full engraftment in this setting (17). Acknowledging these limitations, it is thus important to consider the clinical data in light of the supportive preclinical data, where experimental variables can be rigorously controlled. These mechanistic data clearly demonstrate GC effects on donor T cell priming in the gut and the promotion of trafficking to the BM where effective GVL responses are mediated. Given this highly attractive immunological imprinting, and the fact that these patients currently represent a largely incurable cohort, the use of peritransplant GCs in the haplo-SCT should be further explored in prospective randomized phase II clinical trials, ideally in comparison with PT-Cy.

## Methods

Supplemental Methods are available online with this article.

### Mice.

C57BL/6 (B6.WT, H-2^b^, CD45.2^+^), C57BL/6 background Ptprca (B6.SJL, H-2^b^, CD45.1^+^) were purchased from the Jackson Laboratory. B6C3F1 mice (C57BL/6 × C3H, H-2^b/k^) and B6D2F1 mice (C57BL/6 × DBA/2, H-2^b/d^) were purchased from Japan SLC, Charles River Laboratories, or the Jackson Laboratory. Transgenic and KO mice on a B6 background originated as follows: β-actin luciferase–expressing mice (B6^luc+^) were from R. Negrin (Stanford University, Stanford, California, USA), and *GR^fl/fl^*;*Lck-Cre* mice and *GR^fl/fl^* littermates were supplied by J. D. Ashwell (NIH). Mice were bred at Hyogo College of Medicine or Fred Hutchinson Cancer Research Center animal facilities. Mice were housed in sterilized microisolator cages and received acidified autoclaved water after transplantation.

### BM transplantation.

Mice were transplanted after total body irradiation (TBI; x-ray or 137Cs source at 84–110 cGy/min) in 2 separate doses on day –1, followed by the i.v. injection of leukemic cells (1 × 10^6^) where indicated. On the next day, recipients received TCD BM cells (5 × 10^6^) and whole splenocytes (2 × 10^7^) or magnetic beads-purified CD3^+^ T cells (2 × 10^6^ to 5 × 10^6^) from donor mice via the tail vein. Where TCD BM controls were included, cells were prepared using an antibody incubation followed by complement depletion as described previously (10, 11, 28). In some experiments, TEa.Rag1^–/–^ T cells (4000) combined with polyclonal WT T cells (2 × 10^6^) from donor mice were injected concurrently. TBI doses were as follows: B6 mice, 1,000 cGy; B6D2F1 mice, 1,100 cGy; B6C3F1 mice, 1,300 cGy. Recipient mice were injected with water soluble form of dexamethasone (5 mg/kg/day i.p.; Sigma-Aldrich) or vehicle (methyl β-cyclodextrin; Sigma-Aldrich) suspended in PBS solution from days –1 to +5 after BMT.

### Assessment of acute GVHD and leukemic death.

Primary leukemia cells were generated using the expression of the human oncogenes MLL-AF9 or BCR/ABL-NUP98/HOXA9 to model human AML and cryopreserved at disease onset, for subsequent transplantation. Leukemia cells were thawed on the day of injection and included in grafts at 1 × 10^6^/mouse. Mice were scored according to standard protocols (10, 28) and euthanized if clinical score reached ≥ 6 or if weight loss exceeded 30% (which was classified as GVHD death). The mice receiving leukemia were euthanized in accordance with animal ethics guidelines (when WBC count is greater than 50 × 10^6^/mL or 50% of WBC are leukemia as measured by GFP^+^ cells in peripheral blood or when there was evidence of hind limb paralysis). For deaths caused by leukemia, leukemia burden in peripheral blood at either terminal or last routine bleed had to meet the following criteria: greater than 10% GFP^+^ cells in peripheral blood (with any total WBC count) or present at any level but with a total ≥ WBC 10 × 10^6^/mL.

### GVHD histopathology.

Samples of recipient liver and intestines were fixed in 10% formalin, embedded in paraffin, sectioned, mounted on microscope slides, and stained with H&E. Histological images were captured using a Nikon E600 microscope with a Ds-Fi1-U2 digital camera (Nikon). Pathological GVHD scores of the samples were measured by a scoring system, reported by Chen et al. (35). For the immunofluorescence staining, recipient tissues were embedded in compound, followed by snap freezing in a mixture of n-hexane and dry ice. Samples were cut into 4 μm sections, followed by fixation in 3% buffered paraformaldehyde for 1 minute. The sections were preincubated with Block-Ace (DS Pharma) containing 0.1% Triton-X and purified rat anti–mouse CD16/32 Ab (Supplemental Table 4) for 1 hour at room temperature to block nonspecific binding. Optimal dilution of rabbit anti-CD4 (Abcam) and rat anti-CD8a (Abcam) were used for primary staining of the samples, followed by secondary incubation with Alexa 488–conjugated anti–rabbit IgG and Alexa 568–conjugated anti–rat IgG Abs (Abcam), respectively. Slides were mounted with Prolong Gold Antifade Mountant containing DAPI (Thermo Fisher Scientific). Colored images were taken using a Zeiss LSM 780 Confocal Microscope (Carl Zeiss) and Zeiss ZEN software, and images were composed using Adobe Photoshop CS5 (Adobe).

### Flow cytometric analysis.

Cells were incubated with anti-CD16/32 (2.4G2) before antibody staining to block nonspecific binding. The following reagents or anti-mouse mAbs were purchased from BD Biosciences, BioLegend, or Thermo Fisher Scientific, specified in Supplemental Table 4. Cell numbers were counted by cell counter (Horiba). Multicolor flow cytometric analysis was performed by LSR Fortessa X-20 or BD FACSymphony using FACS Diva software (BD Biosciences). FlowJo 9.0 software (Tree Star Inc.) was used for the data analysis; a full list of mAbs utilized is given in Supplemental Table 4. The YAe antibody reacts with Ea peptide (peptides 52–68) bound to I-Ab. Intracellular staining was performed to detect cleaved caspase-3/8 or CXCL12 using BD Cytofix/Cytoperm kit (BD Pharmingen). Simultaneous intracellular staining with FoxP3, Ki-67, or Hoechst dye was performed using an intracellular fixation/permeabilization buffer set (Thermo Fisher Scientific). Zombie Aqua or Zombie NIR fixable viability kit (BioLegend) was used to exclude dead cells. To assess the cell cycle with Ki-67 and Hoechst staining, the cells in the G0, G1, and S/G2/M were defined as Hoechst^lo^Ki-67^lo^, Hoechst^lo^Ki-67^+^, and Hoechst^+^Ki-67^+^, respectively.

### Analysis of cell proliferation in vivo.

To track the division of adoptively transferred cells, magnetic beads-purified donor CD3 T cells (15 × 10^6^) from donor mice were stained with 5 μM violet dye (Tag-It Violet Proliferation Cell Tracking Dye; BioLegend), followed by incubation at 37°C for 10 minutes. The labeling reaction was quenched by washing in PBS supplemented with 5% FBS. Violet dye–labeled CD3^+^ T cells were injected without BM cells. FlowJo software (version 9.6 or 10.7.1) was used for the data analysis.

### In vitro bioluminescent cytotoxic T lymphocytes assays.

Donor-type CD8^+^ T cells (staining for H-2K^d+^TCR-β^+^NK1.1^–^CD44^+^CD8b^+^) were sort-purified from recipient femur BM cells at day 10 after transplantation using FACS Aria III and cocultured with luciferase-expressing leukemic cells (4 × 10^3^/well) in RPMI medium containing mIL-3 together with or without soluble anti-CD3ε mAb (2.5 μg/mL; Thermo Fisher Scientific, 16-0031-82/145-2C11) at the effector/target ratio of 10:1. Cells were then harvested after 4-hour coculture, and an aqueous solution of D-luciferin was added in each culture 10 minutes prior to detection for the photon emission using a luminescence counter (2030 ARVO X, Perkin-Elmer). Photons per second emitted from residual leukemic cells were quantified over 10 seconds in the coculture wells (36, 37). Donor-type CD44^+^CD8^+^ T cells were also cultured for 4 hours at 37°C in RPMI medium containing Brefeldin A. Cultured cells were then permeabilized and intracellular stained for granzyme B granules using BD Cytofix/Cytoperm kit (BD Pharmingen).

### In vivo BLI.

Mice were anesthetized with isoflurane and then injected with D-Luciferin (0.5 mg s.c., PerkinElmer) 5 minutes before each imaging. Photons emitted from luciferase-expressing cells were quantified over a 5-minute exposure using the imaging system (IVIS Spectrum; Perkin Elmer) with Living Image Software. Light emission was presented as photons per second per square centimeter per steer radiant (ph/s/cm^2^/sr), and total flux of each organ, presented as ph/s, was used for comparisons.

### Cell isolation from small intestine and BM.

Terminal ileum was removed, cut longitudinally into 5 mm pieces, and washed 3 times with PBS. The pieces were incubated under stirring in Ca/Mg-free HBSS containing 5 mM EDTA and 10 mM HEPES for 20 minutes at 37°C before thoroughly vortex. Cells were isolated by passing through a 100 μm cell strainer, repeating the procedures 2 times. Suspended cells were kept as the intestinal epithelial cells (IEC). In some experiments, BM cells were flashed and incubated in Ca/Mg^+^ HBSS containing 1 mg/mL collagenase D and DNase 1 (Roche) for 30 minutes at 37°C. Cells were filtered through a 100 μm cell strainer. Suspended cells were analyzed for the BM stroma, including mesenchymal stem cells.

### Intravascular fluorescent Ab staining.

Mice were injected with 3–5 μg of a fluorescent labeled antibody (anti–CD45 PE, 30F-11) by i.v. injection (in 200 μL of PBS) up to 5 minutes before euthanasia. BM cells were collected by flushing femurs (or tibiae) with RPMI + 3% FCS immediately after sacrifice to rinse way any excess Ab (38).

### RNA-Seq on T cells in BM sorted by FACS.

B6D2F1 mice were transplanted with 2 × 10^6^ purified CD3^+^ T cells from B6.CD45.1^+^ mice with or without dexamethasone injection. On day 5 after transplantation, recipient mice were injected with anti–CD45 PE antibody before euthanasia. Single-cell suspensions of PE^+^ fraction on donor-type CD45.1^+^CD8^+^ T cells in the pooled BM cells (2–3 mice/biological group) from the vehicle- and GC-treated recipients were acquired on a Sony MA900 cell sorter (purity at 93%–97%). Total RNA was extracted from each sorted T cell using a RNeasy micro kit (Qiagen), and each sample was evaluated for the RNA integrity by TapeStation system (Agilent). cDNA synthesis and enrichment were performed using the SMART v4 protocol (Takara; Clontech), and library preparation was conducted using an Illumina Nextera XT library preparation kit. The libraries were sequenced using the Illumina NextSeq 2000 platform and a 200 bp paired-end configuration. Raw fastq output was generated from Illumina bcl files using bcl2fastq. Fastqs were aligned to the genome (GRCm39; Gencode M26 annotation; ref. 39) using STAR (40), and read counts were calculated using the Bioconductor function SummarizeOverlaps with default parameters. DESeq2 (41) was used to calculate differentially expressed genes using default parameters, and fold change values were subject to shrinkage using the ashr (42) package. A heatmap was generated using genes that were significantly differentially expressed using a *P* < 0.05, which had been adjusted for multiple hypothesis testing.

### Clinical data source and inclusion criteria.

Forty-four consecutive adult patients with AML in a non-CR state underwent haplo-SCT with a GC-based GVHD prophylaxis using non-TCD PBSCs from an HLA-haploidentical–related donor (2–3 antigen-mismatches in the GVHD direction) in Hyogo College of Medicine Hospital between 2008 and 2017. As for PT-Cy haplo-SCT, the transplant data were obtained from the TRUMP system in Japan. Included in this analysis are adults with AML who received their first allo-SCT using haploidentical donors in non-CR between 2008 and 2014. Inclusion and exclusion criteria were described previously (19). Other clinical data regarding a preparative regimen for GC haplo-SCT, end points and definitions, and statistical analysis are detailed in Supplemental Methods.

### Statistics.

Extensive descriptions of statistical analysis of clinical and preclinical data are supplied in Supplemental Methods. Briefly, the cumulative incidence curves of neutrophil and platelet recovery, acute and chronic GVHDs, relapse, and NRM were estimated with the use of the cumulative incidence function, which accounted for competing risks in the following: for neutrophil or platelet recovery, NRM was a competing event; for acute or chronic GVHD, NRM and relapse were competing events; and for relapse, NRM was a competing event. The cumulative incidence curves were compared using Gray’s test. Overall survival curves were depicted using Kaplan-Meier estimate with log-rank test. All tests of significance were 2-sided, and *P* values less than 0.05 were considered significant. Statistical analyses were performed with EZR, which is a graphical user interface for R. Preclinical data was analyzed utilizing parametric or nonparametric statistics depending on whether data was normally distributed as described in Supplemental Methods.

### Study approvals.

The institutional Review Boards of the Hyogo College of Medicine and the Japan Society for Hematopoietic Cell Transplantation (JSHCT) approved this study. All mice were maintained in our specific pathogen–free facility and treated in accordance with the guidelines for animal care approved by Hyogo College of Medicine and Fred Hutchinson Cancer Research Center. All animal maintenance and experimental procedures were undertaken using protocols approved by each IACUC (Hyogo 17-025, FHCRC 51055 and 51074).

## Author contributions

Conceptualization, design, and writing of manuscript were contributed by GRH, T Inoue, and HO; statistical analysis of clinical data was contributed by TD and HO; recruitment and treatment of patients were contributed by KK, K Ikegame, SM, and SI; clinical data collection was contributed by TF, HN, TA, YM, K Ishiyama, T Ichinohe, and YA; generation and analysis of murine data were contributed by T Inoue, MK, KSE, LS, ST, PZ, SAM, SNF, and BRB; review and writing of manuscript by GRH, T Inoue, MK; and supervision was contributed by GRH, MK, and HO.

## Supplementary Material

Supplemental data

## Version 1 10/12/2021

In-Press Preview

## Version 2 11/22/2021

Electronic publication
