# Supplementary material for: Peritransplant glucocorticoids redistribute donor T cells to the bone marrow and prevent relapse after haploidentical SCT
Source: JCI Insight. 2021 Nov 22;6(22):e153551. doi: 10.1172/jci.insight.153551 (PMC8663779; doi:10.1172/jci.insight.153551)
Supplement: Supplemental data [file jciinsight-6-153551-s130.pdf]

Supplemental Material

Preparative regimen for glucocorticoid-based (GC) haplo-SCT

For patients undergoing glucocorticoid-based (GC) haplo-SCT, the conditioning regimen consisted of fludarabine at 30 mg/m2/day for 6 days (days -9 to -4), melphalan at 70 mg/m^2/day for 2 days (days -3 to -2) and rabbit anti-thymocyte globulin (ATG: thymoglobulin) at 1.25 mg/kg/day for 2 days (days -2 to -1), and total body irradiation at 3 Gy for 1 day (day 0). For the patients who had leukemia cells >10% in the PB and/or BM, cytarabine at 2 g/m^2 for 4 days (days -9 to -6) was added to the conditioning regimen. Peripheral blood stem cell (PBSC) mobilization using granulocyte colony stimulating factor was performed as described previously[1]. T-cell depletion was not performed. PBSCs were freshly transplanted on days 0 and +1. After transplantation, patients received G-CSF from day 5 to the day of neutrophil engraftment. GVHD prophylaxis consisted of methylprednisolone (mPSL) and tacrolimus. mPSL injection was started at a dose of 2 mg/kg on days -2 and -1 (on days of ATG administration), increased further to 500 mg/day on days of stem cell infusion (on days 0 and 1), and thereafter resumed at 1 mg/kg in divided doses twice a day. Dose reductions in mPSL were commenced on day 15 and undertaken rapidly until day 30 (the target dose on day 30 was 0.5 mg/kg/day). Continuous intravenous administration of tacrolimus was started at a dose of 0.02 mg/kg/day from day -2. The target blood concentration of tacrolimus was 8 – 12 ng/mL up to day 30, and thereafter tapered in the absence of acute GVHD. Patients received intravenous tacrolimus therapy until they could reliably receive oral medications after transplantation. Diagnosis and treatment of acute or chronic graft-versus-host disease, and supportive care were previously described[1].

End points and definitions

The primary end point was overall survival (OS), which was defined as time from transplantation to death from any cause. Relapse was defined as a recurrence or progression of underlying

hematologic malignant diseases. Patients who did not achieve a CR after allo-SCT were considered as having a relapse on day 1 in the analysis of relapse. Non-relapse mortality (NRM) was defined as death without relapse or disease progression. Observation times were censored at the date the patient was last seen alive in case the event of interest was not observed. Neutrophil recovery was defined as achieving absolute neutrophil count ≥ 0.5 x 10^9/L for 3 consecutive days, and platelet recovery defined as achieving platelet count ≥ 2.0 x 10^9/L for at least 7 days, unsupported by transfusion.

Statistical analysis of clinical data

Continuous and categorical data are summarized as medians with ranges (minimums – maximums) and as frequencies with percentages, respectively. In our recent study, we showed that leukemic burden before transplantation was an independent risk factor for patients with AML who underwent allogeneic SCT in non-CR state[2]. Specifically, we reasonably categorized patients with AML who underwent allo-SCT in non-CR, into one of three groups according to leukemic burden, 1) BM blast ≤ 20% with the absence of PB blasts, 2) BM blast ≤ 20% with the presence of PB blasts, or 20% < BM blast ≤ 60%, or 3) BM blast > 60%, referred to leukemic burden levels A, B, and C, respectively. In the multivariable Cox proportional-hazards model, continuous variables, such as patient age, were categorized into one of three groups for ease of interpretation, ≥ 15 to ≤ 40, > 40 to ≤ 50, or > 50 to ≤ 70 under the hazard ratios of the categories. For the other categories, the patients were categorized into one of the following groups: donor-recipient sex combination (female donor to male recipient, versus all others), stem cell source GC–haplo-SCT versus PT-Cy–haplo-SCT), cytogenetics (good/ intermediate karyotype or poor karyotype), time interval from diagnosis to transplant (< 12 months versus ≥ 12 months), HCT-CI (0 versus ≥ 1), and disease status (relapse 1, relapse 2, or PIF). As melphalan 140 mg/m^2 used in our regimen was mid-intensity[3], we excluded this variable from the prognostic factors for the multivariable Cox proportional-hazards model. The

cumulative incidence curves of neutrophil and platelet recovery, acute and chronic GVHDs, relapse, and NRM were estimated with the use of the cumulative incidence function, which accounted for competing risks in the following: for neutrophil or platelet recovery, NRM was a competing event; for acute or chronic GVHD, NRM and relapse were competing events; and for relapse, NRM was a competing event. The cumulative incidence curves were compared using Gray's test. Overall survival curves were depicted using Kaplan-Meier estimate with log-rank test. The impact of GVHD prophylaxis using either GC or PT-Cy on each of the outcomes, *i.e.*, relapse, NRM, neutrophil and platelet recovery, and acute and chronic GVHD, in which competing risks should be accounted, was evaluated with the use of a multivariable Fine and Gray proportional-hazards model[4] with adjustment for the same prognostic factors as in the multivariable Cox proportional-hazards model. All tests of significance were two-sided, and *p*-values less than .05 were considered significant. Statistical analyses were performed with EZR[5,6] which is a graphical user interface for R.

Statistical analysis of pre-clinical data

Statistical analysis was performed on Prism version 8.0 or 9.0 software (Graphpad Software). If the data was normally distributed, unpaired 2-tailed Student's t-test with Welch's modification was used to compare the two groups. Dunnett T3 test after one-way Welch's ANOVA was used for multiple test comparisons. For the data that was not normally distributed, Dunn's test after nonparametric Kruskal-Wallis test was used for multiple group comparisons or nonparametric Mann-Whitney U test (two-tailed) for comparisons of two groups. Survival curves were plotted using Kaplan–Meier estimates and compared by log-rank analysis. R software was used to analyze the survival curve where the leukemia death accounted for a competing risk in the GVHD mortality after transplantation. Data are presented as the mean ± SEM and $P < 0.05$ considered significant. GraphPad Prism 8 was used for the generation of graphs and for statistical analysis. *, $P < 0.05$; **, $P < 0.01$; ***, $P < 0.001$; ****, $P < 0.0001$.

References

1. Ogawa H, Ikegame K, Yoshihara S, et al. Unmanipulated HLA 2-3 antigen-mismatched (haploidentical) stem cell transplantation using nonmyeloablative conditioning. *Biol Blood Marrow Transplant.* 2006;12(10):1073-1084.
2. Ikegame K, Yoshida T, Yoshihara S, et al. Unmanipulated Haploidentical Reduced-Intensity Stem Cell Transplantation Using Fludarabine, Busulfan, Low-Dose Antithymocyte Globulin, and Steroids for Patients in Non-Complete Remission or at High Risk of Relapse: A Prospective Multicenter Phase I/II Study in Japan. *Biol Blood Marrow Transplant.* 2015;21(8):1495-1505.
3. Giralt S, Ballen K, Rizzo D, et al. Reduced-intensity conditioning regimen workshop: defining the dose spectrum. Report of a workshop convened by the center for international blood and marrow transplant research. *Biol Blood Marrow Transplant.* 2009;15(3):367-369.
4. Fine JP, Gray RJ. A Proportional Hazards Model for the Subdistribution of a Competing Risk. *Journal of the American Statistical Association.* 1999;94(446):496-509.
5. Kanda J. Scripts for TRUMP data analyses. Part II (HLA-related data): statistical analyses specific for hematopoietic stem cell transplantation. *Int J Hematol.* 2016;103(1):11-19.
6. Kanda Y. Investigation of the freely available easy-to-use software 'EZR' for medical statistics. *Bone Marrow Transplant.* 2013;48(3):452-458.

Supplemental Table 1. Patients characteristics

		GC haplo	PT-Cy haplo
Number of patients		44	29
Sex	male/female	27/17	15/14
Age (y)	median (range)	53 (31-65)	49 (16-66)
Disease status	primary induction failure	29	19
	first relapse	12	9
	second relapse	3	1
Donor	offspring	31	13
	sibling	9	11
	parent	1	4
	others	3	1
HLA disparity in GVH direction	1 antigen	0	2
	2 antigens	24	14
	3 antigens	20	13
Karyotype	good	4	4
	intermediate	26	15
	poor	13	10
	unknown	1	0
HCT-CI	0	18	13
	1	4	5
	2≤	22	11
Conditioning regimen	Flu+(CA)+L-PAM +TBI(3)+ATG	44	0
	Flu+BU+TBI±others	0	19
	Flu+TBI	0	4
	Flu+BU+CA+ATG	0	5
	others	0	1
GVHD prophylaxis	Tac+mPSL	44	0
	Tac+MMF	0	17
	CSP+MMF	0	3
	Tac±MTX	0	8
	MMF	0	1
Stem cell	BM	0	0
	PB	44	29
	CD34 cells (x10^6/kg)	6.08 (1.91-11.56)	2.53 (0.14-5.86)
Leukemic burden	[a]Level A	7 (15.6)	6 (20.7)
	[b]Level B	15 (34.1)	11 (37.9)
	[c]Level C	22 (50.0)	12 (41.4)
Blast (%) in the BM	median (range)	44.1 (0-93)	36.8 (0-95)
Blast (%) in the PB	median (range)	22.8 (0-99)	7.0 (0-90)

[a]Level A, BM blast ≤ 20% and the absence of PB blasts; [b]Level B, BM blast ≤ 20% and the presence of PB blasts or 20%< BM blast ≤ 60%; [c]Level C, BM blast > 60%

Supplemental Table 2. Multivariable analysis of outcome

	GC haplo (versus PT-Cy haplo)	
	HR (95%CI)	*p*-value
Relapse	0.279 (0.121-0.645)	0.003
Non-relapse mortality	3.797 (0.581-24.800)	0.160
Acute GVHD	3.192 (1.019-9.994)	0.046
Chronic GVHD	3.315 (0.420-2.618)	0.260
Neutrophil recovery	11.170 (5.480-22.760)	< 0.001
Platelet recovery	5.178 (2.750-9.753)	< 0.001

Supplemental Table 3. Multivariable analysis for mortality

	Overall mortality	
Variables	HR (95%CI)	*p*-value
Leukemic burden		
Level A[a]	1.00	
Level B[b]	0.869 (0.307-2.459)	0.791
Level C[c]	0.473 (0.153-1.466)	0.195
Donor-recipient sex combination		
All other	1.00	
Female donor, male recipient	0.508 (0.190-1.361)	0.178
GVHD prophylaxis		
PT-Cy haplo	1.00	
GC haplo	0.376 (0.148-0.960)	0.041
Cytogenetics		
Good/Intermediate	1.00	
Poor	1.392 (0.568-3.409)	0.470
Recipient age (years)		
15-40	1.00	
41-50	1.512 (0.366-6.246)	0.568
51-70	2.823 (0.850-9.376)	0.090
HCT-CI		
1 ≤	1.00	
0	1.370 (0.614-3.060)	0.442
Time interval from diagnosis to transplant		
≥ 12 months	1.00	
< 12 months	1.308 (0.357-4.794)	0.686
Disease status		
PIF	1.00	
First relapse	1.169 (0.398-3.434)	0.776
Second relapse	4.370 (0.723-26.430)	0.108
Years at allo-SCT	0.951 (0.734-1.233)	0.706

Abbreviations: HR, hazard ratio

[a]Level A, BM blast ≤ 20% and the absence of PB blasts; [b]Level B, BM blast ≤ 20% and the presence of PB blasts or 20%< BM blast ≤ 60%; [c]Level C, BM blast > 60%

Supplemental Table 4. Antibodies and staining dyes

Antigen	Conjugated fluorochrome	Clone	Catalog number	Supplier
**Intracellular staining**				
active Caspase 3	PE	C92-605	550821	BD Biosciences
Caspase 8 (CaspGLOW kit)	FITC		88-7005	Thermo Fisher
Ki67	APC	16A8	652405	BioLegend
CXCL-12	FITC	79018	IC350F	R&D Systems
**Surface staining**				
CD3e	PECy7	145-2C11	100320	Biolegend
CD4	APC	RM4-5	553051	BD Biosciences
CD4	AF700	GK1.5	100430	Biolegend
CD8a	BV711	53-6.7	100748	Biolegend
CD8a	BV650	53-6.7	100742	Biolegend
CD8a	PB	53-6.7	100725	Biolegend
CD90.1	APCeFluor780	HIS51	47-0900-82	eBioscience
CD90.2	BV605	53-2.1	140318	Biolegend
TCRβ	FITC	H57-597	109206	Biolegend
CD44	APCCy7	IM7	560568	BD Biosciences
CD62L	BV480	MEL-14	746726	BD Bioscience
CD103	PE	M290	557495	BD Biosciences
CD103	BV421	M290	562771	BD Bioscience
CXCR4	PerCPCy5.5	L276F12	146510	Biolegend
α4 β7 integrin	PE	DATK32	120606	Biolegend
α4 integrin (CD49d)	PEDazzle594	R1-2	103626	Biolegend
β1 integrin (CD29)	BV650	HM b1-1	740500	BD Bioscience
VCAM-1 (CD106)	PE	429 (MVCAM.A)	105714	Biolegend
Vα2 TCR	APCCy7	B20.1	127818	Biolegend
Vβ6 TCR	FITC	RR4-7	553193	BD Bioscience
I-A/I-E	FITC	M5/114.15.2	107606	Biolegend
I-A/I-E	PB	M5/114.15.2	107620	Biolegend
CD64	PECy7	X54-5/7.1	139314	Biolegend
CD11c	APCCy7	N418	117324	Biolegend
YAe	Biotin	eBioY-Ae	13-5741-85	eBioscience
Streptavidin	BV421		405226	Biolegend
Streptavidin	BV605		405229	Biolegend
Ly6C	PEDazzle594	AL-21	128044	Biolegend
Ly6G	Biotin	1A8	127604	Biolegend
CD31	PeCy7	MEC13.3	102524	Biolegend
Ter119	APC	Ter119	116212	Biolegend
Sca-1	BV421	D7	108128	Biolegend
CD16/32	purified	2.4G2	553142	BD Bioscience
CD45	PE	30-F11	103106	Biolegend
CD45.1	FITC	A20	110706	Biolegend
CD45.1	AF700	A20	110724	Biolegend
CD45.1	PECy7	A20	110730	Biolegend
CD45.2	FITC	104	109806	Biolegend
CD45.2	APC	104	109804	Biolegend
CD326 (EpCAM)	AF647	G8.8	118212	Biolegend
H2Dd	PE	34-2-12	110608	Biolegend
H2Dd	AF647	34-2-12	110612	Biolegend
H2Kd	AF700	SF1-1.1	116628	Biolegend
**Staining dye**				
Zombie Aqua™ Fixable Viability Kit			423102	BioLegend
Zombie NIR™ Fixable Viability Kit			77184	BioLegend
7AAD			A9400	Sigma-Aldrich
Violet dye cell trace proliferation kit			C34557	BioLegend
Hoechst 33342			561908	BD Biosciences

Supplementary Figures

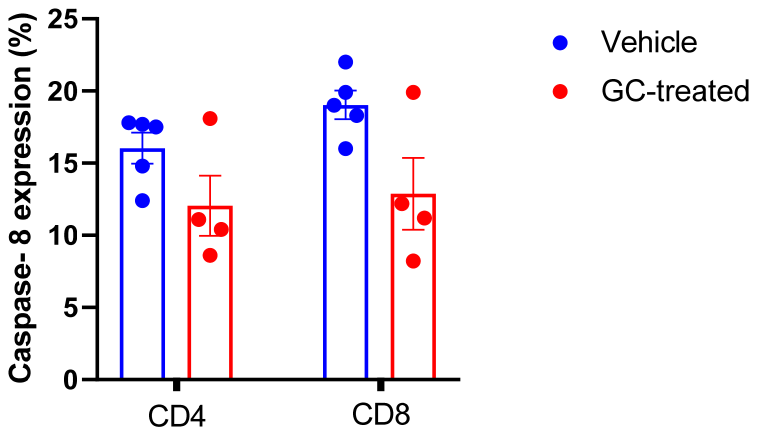

Supplemental Figure 1. Caspase-8 expression of polyclonal donor T cells in mLN at day 5 after SCT. Caspase 8 was quantified in donor T cells within mLN 5 days after SCT by flow cytometry.

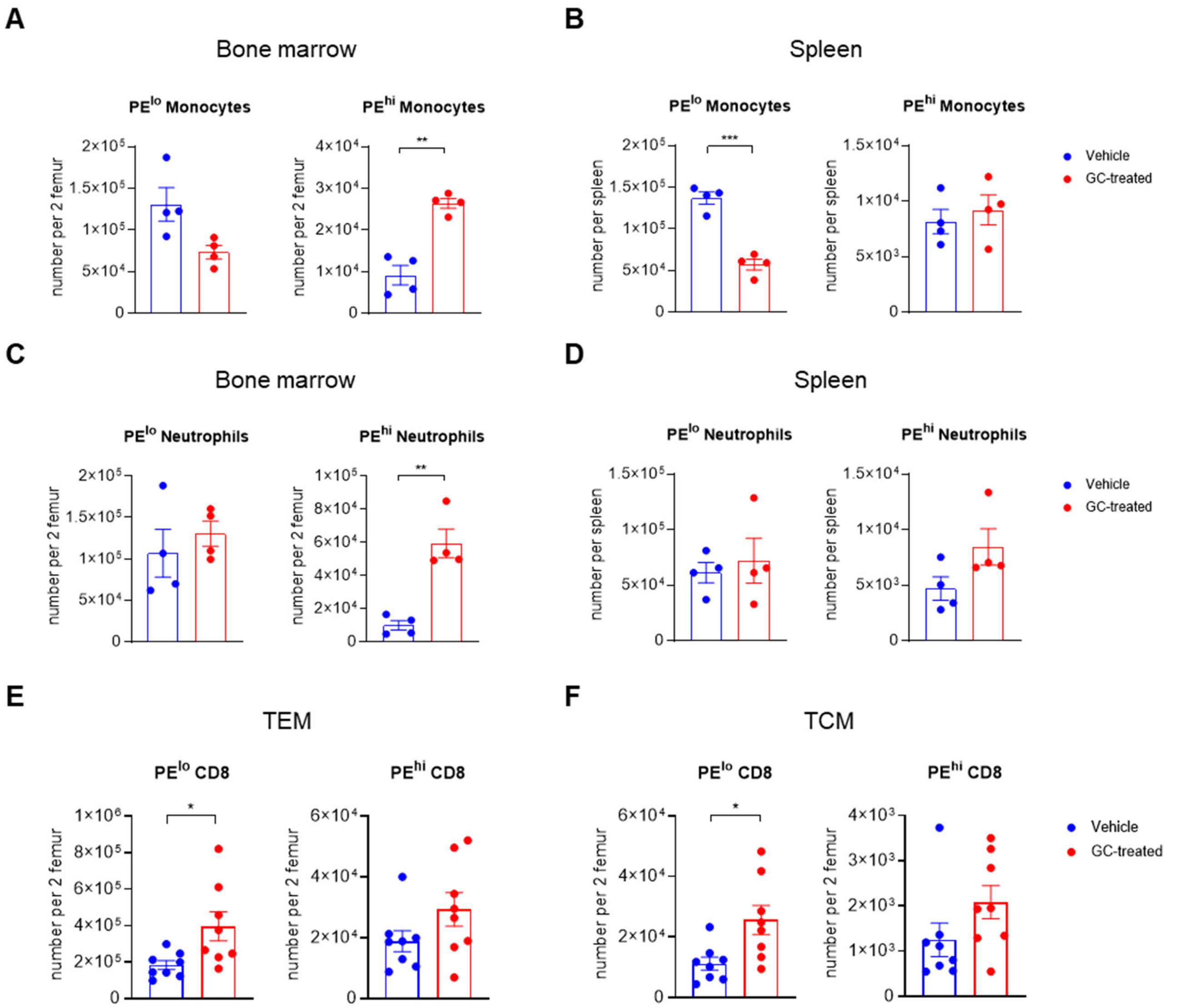

Supplemental Figure 2. Effect of glucocorticoids on cell migration. Recipient mice were injected with PE conjugated anti-CD45 antibody (PE) 5 min before euthanasia. At day 5 after BMT, the absolute numbers of CD45.1$^+$CD45.2^{neg} resident (PE^{neg}) and circulatory (PE$^+$) Ly6C$^+$ monocytes (**A – B**) or Ly6G$^+$ neutrophils (**C – D**) were quantified in the BM (**A, C**) and spleen (**B, D**) (n = 4 – 5 per group). (**E – F**) The absolute numbers of resident (PE^{low}) and circulatory (PE^{high}) CD8 T cells with (**E**) CD44$^+$CD62L^{neg} TEM and (**F**) CD44$^+$CD62L$^+$ TCM phenotypes in the bone marrow 27 days after BMT (n = 7 – 8 per group). Data are presented as mean ± SEM. *P < .05; **P < .01; ***P < .001.

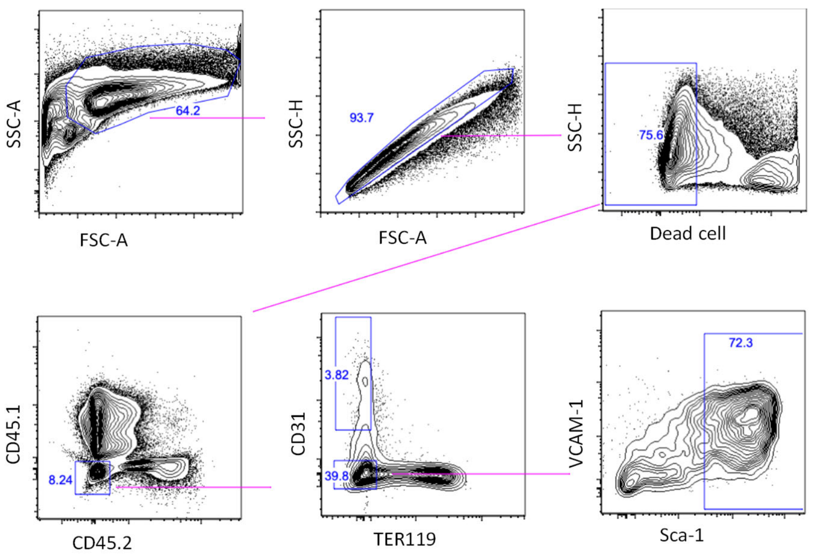

Supplemental Figure 3. Gating of bone marrow stroma. Representative gating strategy for the expressions of CD31^{+}TER119^{neg} endothelial cells and CD45^{neg}CD31^{neg}TER119 ^{neg}Sca-1^{+} mesenchymal stromal cells (MSCs) in the BM at day 5 after BMT.

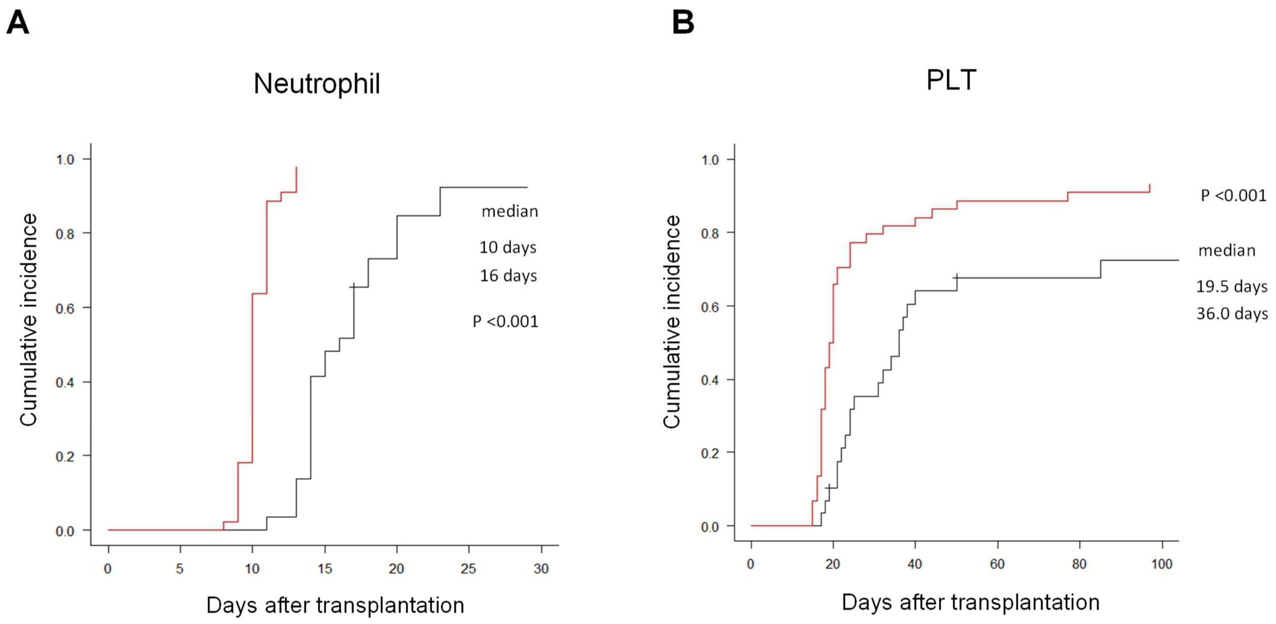

Supplemental Figure 4. Time to engraftment. Donor engraftment of (**A**) neutrophils and (**B**) platelets after haploidentical SCT (red = glucocorticoid-treated, black = PT-Cy).
